# Cra and cAMP Receptor Protein Have Opposing Roles in the Regulation of *fruB* in Vibrio cholerae

**DOI:** 10.1128/JB.00044-21

**Published:** 2021-04-21

**Authors:** Christina Beck, Sayde Perry, Daniel M. Stoebel, Jane M. Liu

**Affiliations:** a Department of Chemistry, Pomona College, Claremont, California, USA; b Department of Biology, Harvey Mudd College, Claremont, California, USA; Queen Mary University of London

**Keywords:** CRP, Cra, FPr, FruR, PTS, *Vibrio cholerae*, fructose

## Abstract

The Gram-negative bacterium Vibrio cholerae adapts to changes in the environment by selectively producing the necessary machinery to take up and metabolize available carbohydrates. The import of fructose by the fructose-specific phosphoenolpyruvate (PEP) phosphotransferase system (PTS) is of particular interest because of its putative connection to cholera pathogenesis and persistence. Here, we describe the expression and regulation of *fruB*, which encodes an EIIA-FPr fusion protein as part of the fructose-specific PTS in V. cholerae. Using a series of transcriptional reporter fusions and additional biochemical and genetic assays, we identified Cra (catabolite repressor/activator) and cAMP receptor protein (CRP) as regulators of *fruB* expression and determined that this regulation is dependent upon the presence or absence of PTS sugars. Cra functions as a repressor, downregulating *fruB* expression in the absence of fructose when components of PTS^Fru^ are not needed. CRP functions as an activator of *fruB* expression. We also report that Cra and CRP can affect *fruB* expression independently; however, CRP can modulate *cra* expression in the presence of fructose and glucose. Evidence from this work provides the foundation for continued investigations into PTS^Fru^ and its relationship to the V. cholerae life cycle.

**IMPORTANCE** Vibrio cholerae is the causative agent of cholera disease. While current treatments of care are accessible, we still lack an understanding of the molecular mechanisms that allow V. cholerae to survive in both aquatic reservoirs and the human small intestine, where pathogenesis occurs. Central to V. cholerae’s survival is its ability to use available carbon sources. Here, we investigate the regulation of *fruB*, which encodes a protein central to the import and metabolism of fructose. We show that *fruB* expression is controlled by the transcriptional regulators Cra and CRP. This work contributes toward a clearer understanding of how carbon source availability impacts the physiology and, potentially, the persistence of the pathogen.

## Abstract (precis)

Vibrio cholerae is the causative agent of cholera disease. While current treatments of care are accessible, we still lack an understanding of the molecular mechanisms that allow V. cholerae to survive in both aquatic reservoirs and the human small intestine, where pathogenesis occurs.

## INTRODUCTION

Caused by the facultative pathogen Vibrio cholerae, cholera disease is estimated to affect three to five million people each year and is characterized by profuse, watery diarrhea, resultant dehydration, and hypovolemic shock (1, 2). While current intravenous and oral rehydration treatments are effective in treating the disease, approximately 1.3 billion people across 51 countries are still at risk for infection due to gaps in health infrastructure (1, 3, 4). The transmission of V. cholerae is most frequently attributed to malfunctioning or inadequate sanitation systems, as well as the lack of clean water sources (1, 3). Important to this transmission is the ability of V. cholerae to adapt to conditions in both the human small intestine, where pathogenesis occurs, and aquatic reservoirs, where the bacteria spread between contaminated water sources (5, 6). In order to survive in both niches, V. cholerae must sense the carbon sources currently available and produce the necessary metabolic machinery to convert available carbon sources to utilizable energy currency (7). The phosphoenolpyruvate (PEP) phosphotransferase system (PTS), a phosphotransfer cascade conserved across many bacterial species and responsible for carbon uptake and phosphorylation for downstream metabolism, is thought to play a role in V. cholerae’s ability to survive in multiple environments (7).

The PTS phosphocascade begins with the transfer of a phosphate group from PEP to EI, a constitutively expressed cytoplasmic protein (8, 9). Next, the phosphate group is passed from EI to histidine protein (HPr or the HPr homolog FPr), another cytoplasmic component (8, 9). From HPr or FPr, the phosphate group is then passed to a number of carbohydrate-specific domains, referred to as EIIA and EIIB (8, 9). Intake of the carbohydrate across the membrane then occurs via the EIIC domain, a carbohydrate-specific transmembrane protein (8, 9). The carbohydrate is concomitantly phosphorylated with this transport step (8, 9). Because carbon specificity lies within the domains of the EII protein, bacteria often have multiple EII proteins, one or more for each carbon source which enters the cell through the PTS (9). Moreover, synthesis of EII proteins is typically induced in the presence of its respective carbon source (8). V. cholerae contains 25 PTS components, including 13 distinct EIIC domains (10). Recently, the carbohydrate specificity of each EIIC transporter was defined; together, the 13 proteins are able to transport fructose, GlcNAc, (GlcN)_2_, glucose, mannitol, mannose, MurNAc, and sucrose into the cell via the PTS (11).

Here, we focus on one of these PTS sugars, fructose. V. cholerae is entirely dependent on the PTS system for the uptake of fructose, in contrast to the existence of multiple non-PTS glucose transporters (7, 11). PTS^Fru^-specific components in V. cholerae are encoded by genes located at two distinct loci. First, VC1826 encodes an EIIABC fusion protein that is capable of both fructose and mannose transport (7, 11). VCA0516, VCA0517, and VCA0518 make up the second locus encoding PTS^Fru^ proteins (12). VCA0518 (*fruB*) encodes the fructose-specific EIIA domain and FPr, which exist as fusion proteins. VCA0517 (*fruK*) encodes 1-phosphofructokinase, which is responsible for phosphorylating fructose-1-phosphate to fructose-1,6-bisphosphate following uptake and phosphorylation of fructose into the cell. VCA0516 (*fruA*) encodes the fructose-specific EIIB and EIIC domains, which exist as fusion proteins. Experimental evidence suggests that in V. cholerae, the proteins encoded by *fruBKA* play the primary role in fructose transport and that FPr is preferred over HPr in PTS transport and phosphorylation of fructose (7, 11).

Relevant to the life cycle of V. cholerae, *fruB* (encoding EIIA-FPr, herein shorted to FPr), along with other PTS components, is upregulated when the bacteria enter their viable but nonculturable state, suggesting that the uptake of specific carbon sources may be important for the survival of these bacteria (13). *fruB* is also induced during infection of a mouse model of cholera, and Δ*fruB* mutants demonstrated a 3-fold defect during a colonization assay of infant mice (14). FPr and HPr, moreover, are involved in a signaling cascade that allows the phosphorylation state of EI to impact biofilm formation in growth conditions involving glucose (7). Thus, FPr, along with other PTS^Fru^ components, may be particularly important as V. cholerae transitions between environments containing high or low concentrations of fructose. A clearer understanding of *fruB* expression and its regulation in response to changes in carbon source availability would shed light on the persistence of the pathogen.

Here, we investigated the roles of two global transcription factors, Cra and cAMP receptor protein (CRP), in regulating *fruB* expression in V. cholerae in various carbon sources. We demonstrate that Cra represses *fruB* transcription in the absence of fructose, likely by acting near the −10 hexamer of the *fruB* promoter, while CRP activates *fruB* expression in the absence of glucose, working farther upstream in the promoter. Our data indicate that the two regulators can work independently to control the production of FPr depending on carbon source availability, although CRP can repress *cra* expression in some growth conditions.

## RESULTS

### Identification of the *fruB* transcription start site.

**FIG 1:**
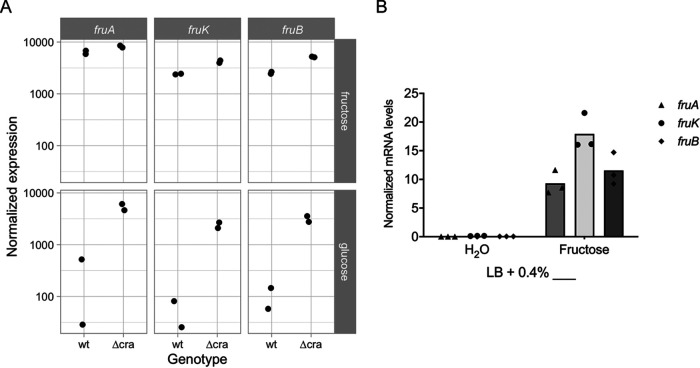
*fruBKA* expression is induced by fructose. (A) Normalized counts for *fruA*, *fruK*, and *fruB* transcripts in wild-type (wt) and Δ*cra* strains in fructose and glucose growth conditions. RNA for RNA-Seq was extracted from bacteria cultured in 1× M9 supplemented with 0.4% (wt/vol) fructose or glucose and grown to an OD_600_ of ∼0.3. (B) *fruA*, *fruK*, and *fruB* expression in a wild-type strain grown in LB supplemented with 0.4% (wt/vol) fructose or an equivalent volume of water. Cultures were grown to an OD_600_ of ∼0.3, and then RNA was extracted, purified, and treated with DNase I. qRT-PCR was performed on each total RNA sample using SYBR green and gene-specific primers. RNA amounts were determined using standard curves and then normalized to an endogenous control (4.5S RNA). Bars represent means from biological replicates.

Before beginning to dissect how *fruB* is regulated, we first confirmed that *fruB*, *fruK*, and *fruA* are all induced by the presence of fructose using a combination of transcriptome sequencing (RNA-Seq) and quantitative reverse transcription-PCR (qRT-PCR). RNA for RNA-Seq was extracted from wild-type (WT) V. cholerae cultured in 1× M9 medium supplemented with either fructose or glucose. As expected, given the locus encodes components of PTS^Fru^, *fruB*, *fruK*, and *fruA*, transcript levels were all higher in the presence of fructose than glucose (Fig. 1A). Moreover, normalized expression of each gene in the *fru* locus showed similar levels of upregulation in fructose as glucose growth conditions, which could suggest coregulation of the three genes. To confirm these results, *fruBKA* transcript levels were also investigated by qRT-PCR. Total RNA was extracted from a WT strain cultured in Luria-Bertani (LB) broth only or LB broth supplemented with fructose. Similar to our RNA-Seq results, all three transcripts were expressed at high levels in fructose medium, and transcript levels decreased in the absence of fructose (Fig. 1B). From both RNA-Seq and qRT-PCR data, we concluded that *fruB*, *fruK*, and *fruA* are each induced and upregulated in fructose medium. A more thorough analysis of the RNA-Seq data, which was done as part of a larger study, will be presented elsewhere.

**FIG 2:**
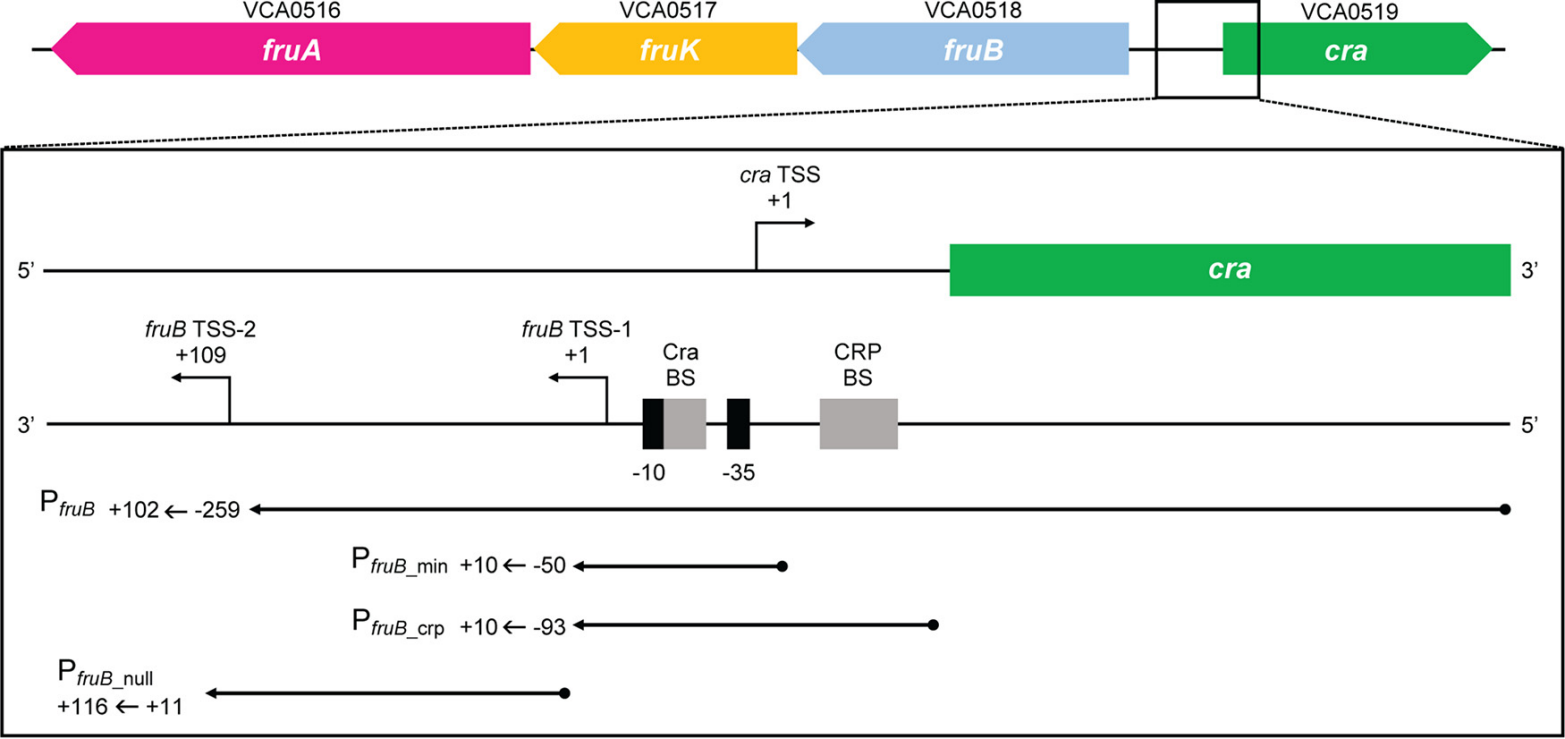
Map of the *fruBKA locus* and *fruB* promoter transcriptional fusions. VCA0518, VCA0517, and VCA0516 (*fruB*, *fruK*, and *fruA,* respectively) encode components of the fructose-specific PTS in V. cholerae. VCA0519 (*cra*) encodes a putative transcriptional repressor. The *fruB* transcription start site (TSS) determined by Papenfort et al. is labeled as *fruB* TSS-1 and was designated the +1 site (16). Based on this numbering scheme, the *fruB* start codon begins at position +242. *fruB* TSS-2 was determined by 5′ RACE in this work (see Fig. S1 and S2 in the supplemental material). P*_fruB_*, P*_fruB_*__min_, P*_fruB_*__crp_, and P*_fruB_*__null_ reporters contain portions of the *fruB* promoter indicated by single-headed arrows fused to the 5′ end of the E. coli *lacZ* gene. Reporters were then inserted into the *lacZ* gene in the V. cholerae genome to disrupt endogenous *lacZ* expression. Exact coordinates included in each fusion are listed next to the respective arrow. Putative −10 and −35 sites in the *fruB* promoter are depicted by black bars. Putative binding sites (BSs) for Cra and CRP are depicted by gray bars.

We then set out to determine the transcription start site (TSS) of *fruB* using 5′ RACE (rapid amplification of cDNA ends) (15). RNA was extracted from a WT strain cultured in minimal medium supplemented with fructose, and a primer annealing to the *fruB* coding region was used to reverse transcribe extracted RNA (see Fig. S1 and Table S2 in the supplemental material). cDNA fragments were then amplified and sequenced. Out of 24 total sequences analyzed across 2 separate 5′ RACE experiments, we observed the *fruB* TSS (notated as *fruB* TSS-2) to lie 133 nucleotides (nt) upstream of the *fruB* start codon in 5 sequences (position +109 relative to TSS-1, described below; Fig. 2; Fig. S1 and S2). In the 19 remaining samples, we were unable to determine the *fruB* TSS because reverse transcription of *fruB*’s 5′ untranslated region (UTR) stopped prematurely downstream of the *fruB* start codon. We also performed 5′ RACE with RNA extracted from a WT strain cultured in minimal medium supplemented with glucose, using the same workflow. In glucose medium, we observed the *fruB* TSS to lie in roughly the same location as in fructose growth conditions, 133 nt upstream of the *fruB* start codon, in 5 of 12 sequences analyzed (Fig. S3). As in fructose medium, failed reverse transcription reactions prevented us from determining the TSS in all 12 samples. The similarities between our 5′ RACE results from fructose and glucose media suggest the location of the *fruB* TSS is not dependent on carbon source when samples are grown in minimal media.

Interestingly, the TSS identified here (*fruB* TSS-2) differs from the TSS identified by Papenfort et al. (notated as *fruB* TSS-1), who reported the V. cholerae *fruB* TSS to be 241 bp upstream of the *fruB* start codon (108 nt upstream of TSS-2) (Fig. 2) (16). In their work, Papenfort and colleagues used differential RNA sequencing (dRNA-Seq) to identify TSSs throughout the V. cholerae genome (16). RNA used in dRNA-Seq was extracted from WT strains cultured in LB without additional carbon sources present (16). It is possible that differences in minimal versus rich media affect the site of transcription initiation. Analysis of our RNA-Seq data points to the 5′ end of *fruB* ranging from roughly the +24 to +74 sites, relative to TSS-1. Therefore, it is also possible that posttranscriptional processing of *fruB* transcripts may occur.

**FIG 3:**
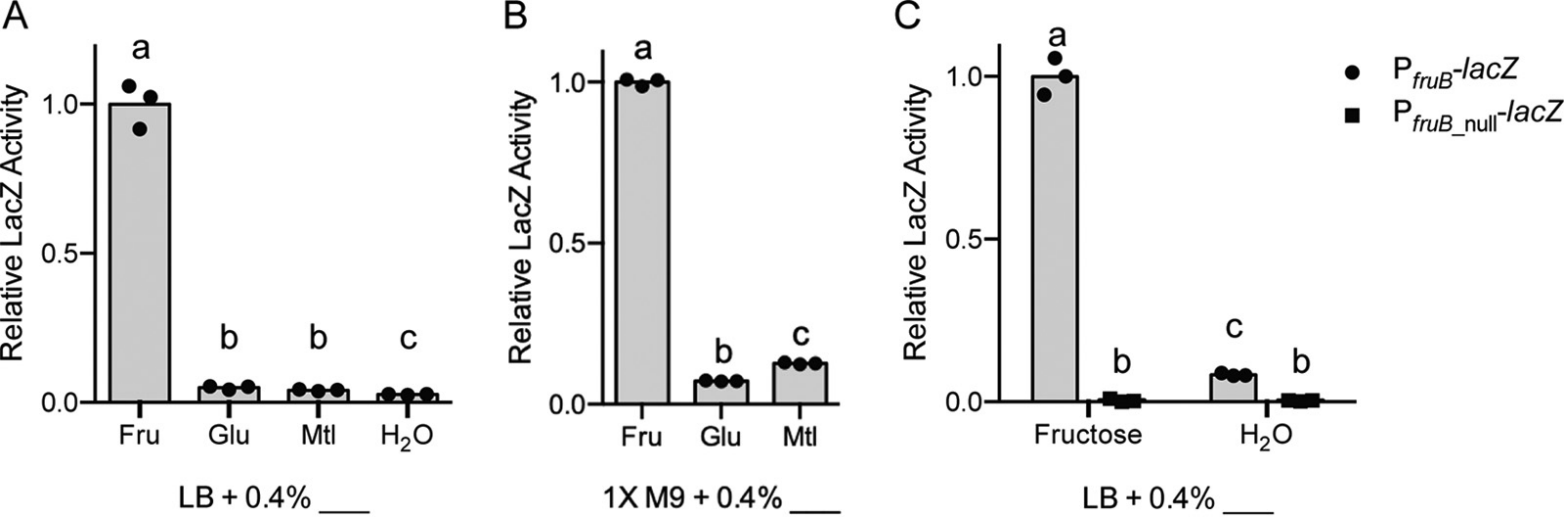
Transcriptional reporter for *fruB* expression. *fruB* promoter activity from the P*_fruB_*-*lacZ* transcriptional reporter (A and B) or P*_fruB_*-*lacZ* and P*_fruB_*__null_-*lacZ* transcriptional reporters (C) in the indicated carbon sources relative to activity from the P*_fruB_*-*lacZ* reporter in fructose conditions (“relative LacZ activity”). Bacteria were initially cultured in LB (A and C) or 1× M9 plus 0.4% (B) maltose overnight and back diluted into fresh media supplemented with the indicated carbon sources the following day. After reaching an OD_600_ of 1.0, cultures were lysed and incubated with ONPG substrate solution for 1 h, from which *A*_420_/minute was measured and normalized to OD_600_. A baseline value was obtained by averaging all three replicates from the P*_fruB_*-*lacZ* reporter in fructose conditions. Relative LacZ activity for other conditions and reporters was calculated by dividing the mean *A*_420_/(minute × OD_600_) value by the baseline, which was set to 1. For each carbon source, cultures were grown in biological triplicate and measured in technical triplicate. Technical triplicates were averaged during data analysis. Bars represent means from biological triplicates. Within each panel, bars with different letters indicate mean values that are significantly different from each other (*P < *0.05, Tukey’s multiple-comparison test).

To reconcile the difference between *fruB* TSS-1 and TSS-2, we constructed the P*_fruB_* transcriptional reporter, which contains a portion of the *fruB* promoter region fused to the 5′ end of the Escherichia coli *lacZ* gene (Fig. 2). This construct was then inserted into the middle of the V. cholerae *lacZ* homolog in the V. cholerae genome to produce the final reporter strain. Through this method, the V. cholerae *lacZ* homolog is rendered inert, and all β-galactosidase activity from the LacZ protein, which reflects transcriptional activity, originates from the promoter region included in the reporter. The portion of the *fruB* promoter included in P*_fruB_* spans the region from −259 to +102, where +1 is *fruB* TSS-1. Notably, the P*_fruB_* reporter includes *fruB* TSS-1 identified by Papenfort and colleagues (i.e., the +1 site) and stops 7 nt before the +1 site of *fruB* TSS-2, which we identified through 5′ RACE (i.e., the +109 site [Fig. 2]). Despite our 5′ RACE data, we postulated that TSS-1 was the more likely start site, as it was the only one of the two that had an identifiable −10 site, which was predicted using BPROM and PromoterHunter and previously determined consensus matrices from both E. coli and V. cholerae (16–18). Using LacZ assays, we then measured transcriptional activity from the P*_fruB_* reporter in a number of PTS carbon sources in both rich (LB) and minimal (M9) media. In both rich and minimal media, we observed the largest amount of β-galactosidase activity (“LacZ activity”) when the medium was supplemented with fructose (Fig. 3A and B). In media supplemented with glucose, mannitol, or, as a control, water, LacZ activity from the P*_fruB_* reporter was statistically lower than with fructose as the added carbon source (*P < *0.05, Tukey’s multiple-comparison test). These results indicate that the P*_fruB_* reporter contains at least one intact TSS, as well as a corresponding transcriptional promoter. The P*_fruB_* reporter must also contain regions responsible for regulation of *fruB* expression, allowing for at least 8-fold (and up to 37-fold) induction in the presence of fructose (Fig. 3A and B).

In addition to the P*_fruB_* reporter, we also constructed the P*_fruB_*__null_ reporter, which spans +11 to +116 of the *fruB* promoter region (Fig. 2). The P*_fruB_*__null_ reporter lacks *fruB* TSS-1 but includes putative −10 and −35 hexamers and the +1 site for *fruB* TSS-2. When we measured LacZ activity, we observed a significant difference in transcriptional activity between the P*_fruB_*__null_ and P*_fruB_* reporters in both of the tested growth conditions (*P < *0.05, Tukey’s multiple-comparison test) (Fig. 3C). Furthermore, no significant difference was observed when comparing transcriptional activity from the null reporter in fructose and water growth conditions. These results suggest the P*_fruB_* reporter contains elements necessary for transcription—and induction in the presence of fructose—while the P*_fruB_*__null_ reporter lacks such sites. We concluded that *fruB* TSS-1, and not *fruB* TSS-2, is the point at which *fruB* transcription begins.

### *fruB* expression is fine-tuned by available carbon sources.

**FIG 4:**
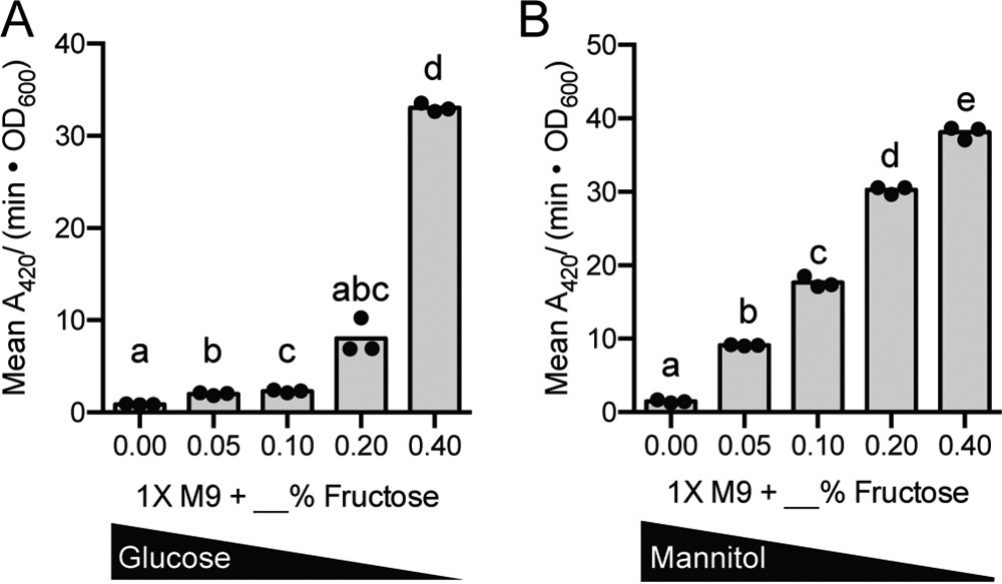
*fruB* expression is carbon source dependent. *fruB* promoter activity in a mixture of carbon sources as measured by mean *A*_420_/(minute × OD_600_) from the P*_fruB_*-*lacZ* transcriptional fusion. Bacteria were initially cultured in 1× M9 plus 0.4% maltose overnight and back diluted into fresh media supplemented with the indicated carbon sources the following day. Cultures were prepared with the indicated percentage (wt/vol) of fructose and brought to a total 0.4% (wt/vol) sugar with additional glucose (A) or mannitol (B). After reaching an OD_600_ of 1.0, cultures were lysed and incubated with ONPG substrate solution for 1 h, from which *A*_420_/minute was measured and normalized to OD_600_. For each carbon source, cultures were grown in biological triplicate and measured in technical triplicate. Technical triplicates were averaged during data analysis. Bars represent means from biological triplicates. Within each panel, bars with different letters indicate mean values that are significantly different from each other (*P < *0.05, Tukey’s multiple-comparison test).

To further probe the dependence of *fruB* expression on the presence of fructose, we conducted LacZ assays with the P*_fruB_* reporter in which cultures were prepared with a mixture of carbon sources, combining fructose and either glucose or mannitol in a range of concentrations. In mixtures of fructose and glucose, LacZ activity from the P*_fruB_* reporter decreased 4-fold when an equal amount of glucose (0.2% wt/vol of 0.4% wt/vol total supplemental sugar) was included in the culture (*P < *0.05, Tukey’s multiple-comparison test) (Fig. 4A). In mixtures of fructose and mannitol, LacZ activity from the P*_fruB_* reporter followed a more linear pattern (Fig. 4B). As a higher percentage of mannitol was included in cultures, LacZ activity significantly decreased (*P < *0.05, Tukey’s multiple-comparison test), although the fold activity reductions were not as high as observed in cultures containing mixtures of fructose and glucose. Similar patterns were observed in analogous Western blots (Fig. S4). These patterns are likely due to the preference for glucose over other carbon sources in bacterial metabolism; in Gram-negative bacteria, carbon catabolite repression regulates metabolic pathways to promote the breakdown of desirable sugars like glucose before others like fructose and mannitol (8, 9, 19). Regardless of carbon source preference, these LacZ assays illustrate the dependence of *fruB* expression on the presence of fructose: a higher percentage of fructose in the system correlates with higher *fruB* expression.

### *fruB* expression is regulated by Cra and CRP.

We next turned our attention to identifying the protein regulators that control induction of *fruB* expression in the presence of fructose. Observations from other Gram-negative bacteria provided a starting point from which to evaluate possible regulators of *fruB* in V. cholerae. Lying directly adjacent to the *fruBKA* locus in V. cholerae, VCA0519 encodes a LacI-GalR family transcriptional regulator referred to both as FruR (fructose repressor) and Cra (catabolite repressor/activator) (Fig. 2) (12). In E. coli, Cra is considered a global transcriptional regulatory protein that affects the metabolism of 36 different carbon sources and represses *fruB* expression (20–24). Alongside Cra, the 3′,5′-cyclic AMP (cAMP) receptor protein (CRP) is another global regulator that affects *fruB* expression in E. coli and Salmonella enterica serovar Typhimurium by activating the gene’s transcription (25, 26). In V. cholerae, moreover, a *crp* mutant strain exhibited decreased expression of *fruB* and *fruA*, along with decreased fructose metabolism (27, 28).

**FIG 5:**
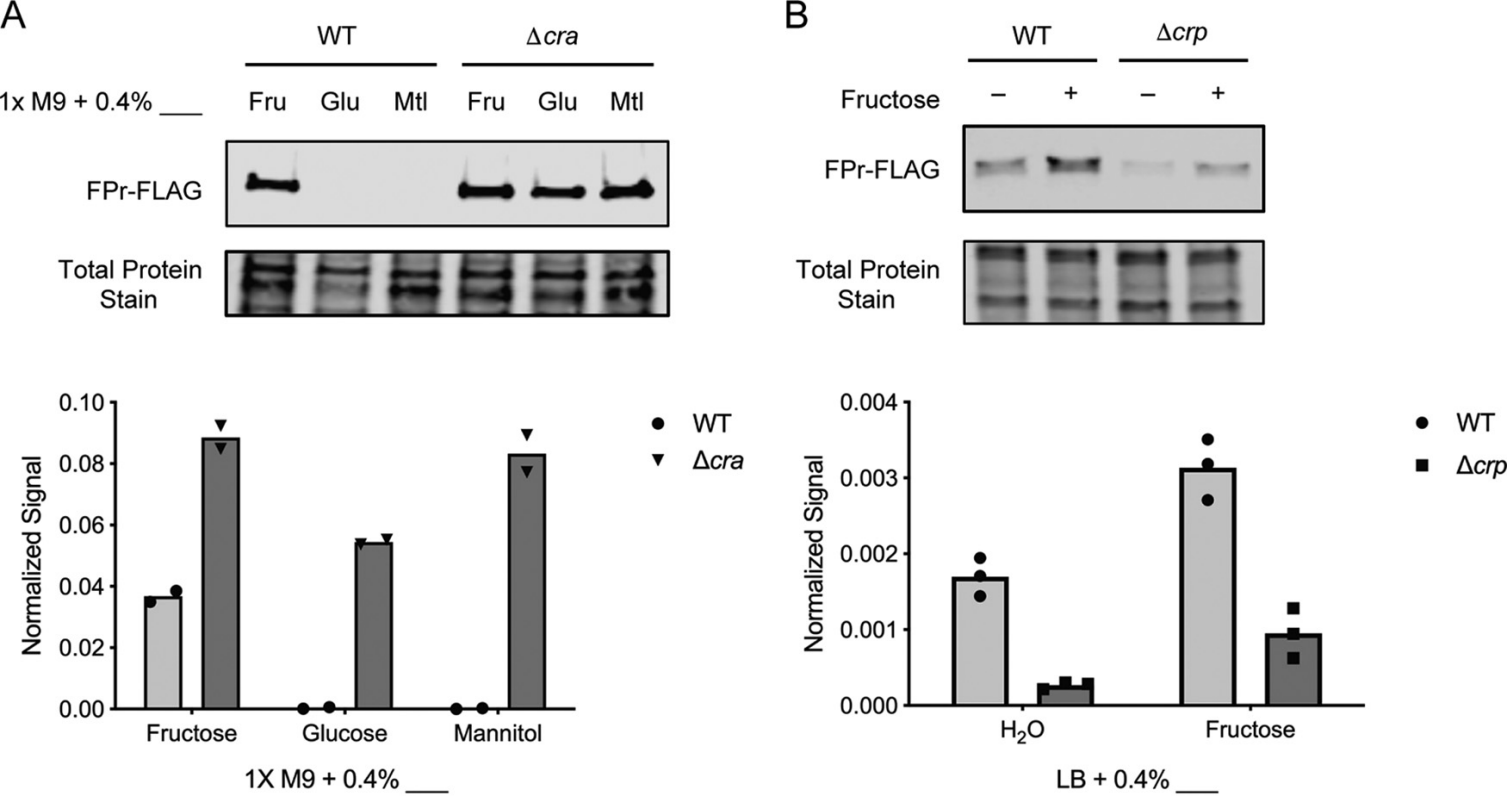
FPr levels are regulated by Cra and CRP. Western blotting of FPr-FLAG in wild-type and Δ*cra* (A) or Δ*crp* (B) strains. Strains were cultured in 1× M9 supplemented with 0.4% (wt/vol) fructose (Fru), glucose (Glu), or mannitol (Mtl) (A) or LB supplemented with 0.4% (wt/vol) fructose (+fructose) or an equivalent volume of water (−fructose) (B). Once cultures reached late log phase, total cell lysate was extracted and run on an SDS-PAGE gel, and anti-FLAG antibodies were used to probe for FLAG-tagged FPr. FPr levels were quantified using LiCor Image Studio and normalized to total protein levels. Bars represent means from biological replicates. In panel B, both pairwise differences comparing WT and Δ*crp* were significant at *P < *0.05 (Sidak’s multiple-comparison test).

We first investigated Cra as a potential regulator of *fruB* in V. cholerae. We repeated our RNA-Seq analysis using a Δ*cra* mutant. In the absence of *cra*, expression of all three transcripts increased in glucose medium, indicating that Cra represses *fruB*, *fruK*, and *fruA* expression in the presence of glucose and providing preliminary evidence that Cra represses *fruB* in nonfructose media (Fig. 1A). To further examine this relationship, we evaluated FPr protein levels in WT and Δ*cra* strains in fructose, glucose, and mannitol growth conditions (Fig. 5A). Similar to the expression patterns observed in our RNA-Seq results, FPr levels were highest in media supplemented with fructose and decreased 100-fold in nonfructose media. In the absence of *cra*, FPr levels increased compared to the WT strain across all culture conditions, supporting the role of Cra as a repressor of *fruB* in nonfructose media. Moreover, these data provide additional evidence that the transcriptional regulator Cra specifically affects *fruB* transcription and such effects are reflected at the protein level as well.

We also evaluated the relationship between CRP and FPr, given evidence which implicates CRP as an activator of *fruB* expression (27, 28). To do so, we measured FPr levels in WT and Δ*crp* strains when LB cultures were supplemented with fructose or an equivalent volume of water (Fig. 5B). Consistent with our previous findings, FPr levels were highest in cultures supplemented with fructose. In the absence of CRP, FPr levels in both fructose and nonfructose conditions decreased 3- to 6-fold (*P < *0.05, Sidak’s multiple-comparison test), suggesting that CRP activates *fruB* expression in both growth conditions.

To confirm that Cra and CRP are regulators of *fruB* transcription, we used the P*_fruB_* reporter to measure *fruB* expression in a series of LacZ assays. In these assays, we measured LacZ activity from the P*_fruB_* reporter in strains lacking either the endogenous *cra* or *crp* loci. In an effort to probe Cra- and CRP-mediated *fruB* regulation independently, these strains (herein referred to as “mutant strains”) also lacked the endogenous locus of the other regulator (i.e., the *cra* mutant strain also lacked the endogenous *crp* locus and was compared to a strain that possessed *cra* but still lacked *crp*). To rule out polar effects, we also measured LacZ activity from a complementation strain that possessed a plasmid containing the *cra* or *crp* gene under the control of an inducible promoter (pJML05::*cra* or pTrc99A::*crp*).

**FIG 6:**
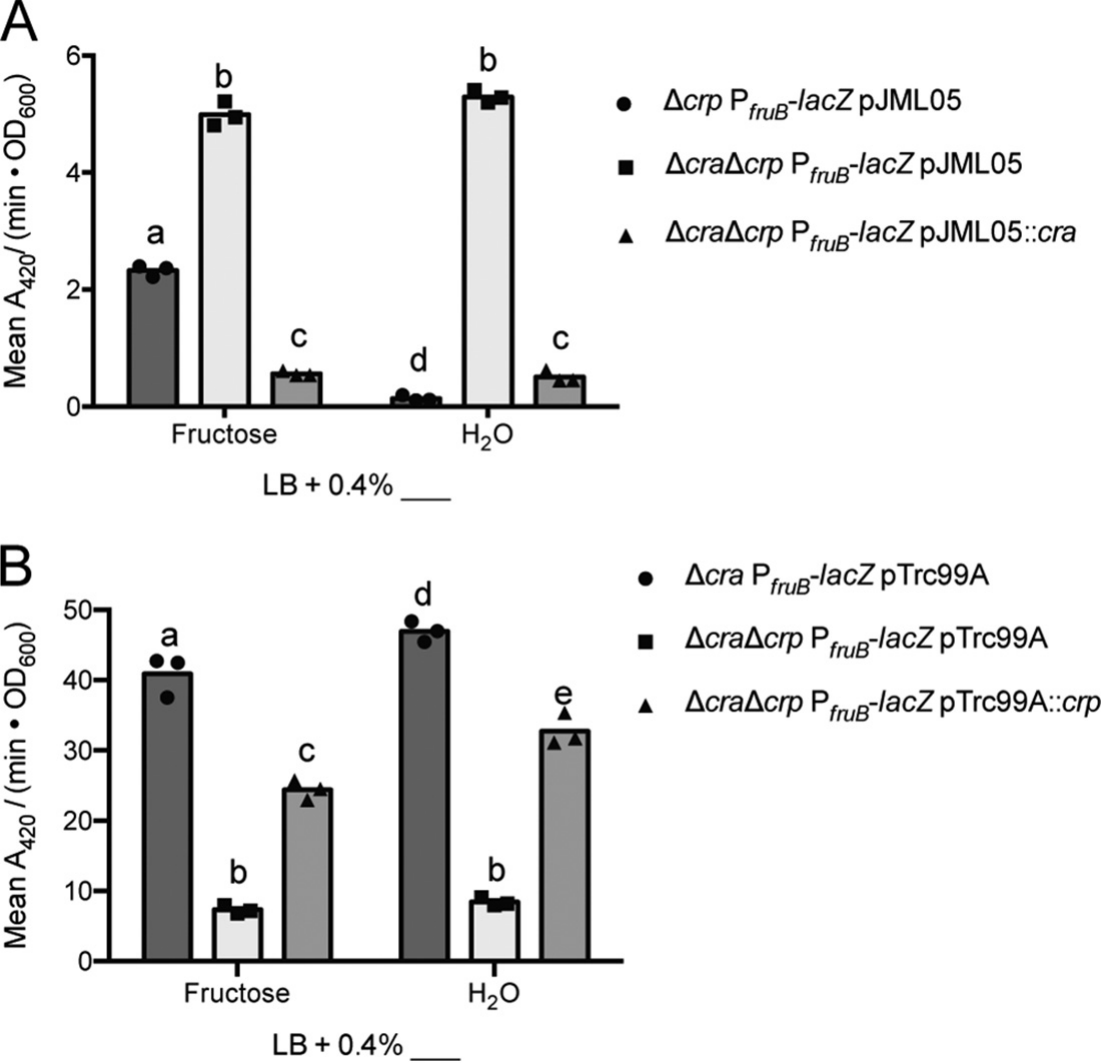
Cra represses *fruB* expression, while CRP activates *fruB* expression. *fruB* promoter activity from the P*_fruB_* transcriptional reporter upon reintroduction of *cra* (A) or *crp* (B) expression via the pJML05::*cra* or pTrc99A::*crp* plasmids or the corresponding empty vectors in Δ*cra*, Δ*crp*, or Δ*cra* Δ*crp* strains. Strains were initially grown solely in LB and back-diluted into fresh cultures supplemented with 0.4% (wt/vol) fructose or an equivalent volume of water. Strains harboring the pJML05 vector or a derivative were also supplemented with 5 mM IPTG, while strains harboring the pTrc99A vector or a derivative were supplemented with 1 mM IPTG. After reaching an OD_600_ of 1.0, cultures were lysed and incubated with ONPG substrate solution for 1 h, from which *A*_420_/minute was measured and normalized to OD_600_. For each carbon source and strain, cultures were grown in biological triplicate and measured in technical triplicate. Technical triplicates were averaged during data analysis. Bars represent means from biological replicates. Within each panel, bars with different letters indicate mean values that are significantly different from each other (*P < *0.05, Tukey’s multiple-comparison test).

In assessing the activity of Cra, the deletion of *cra* resulted in 2- and 38-fold increases in LacZ activity when the strains were grown in fructose and nonfructose media, respectively (*P < *0.05 by Tukey’s multiple-comparison test) (Fig. 6A). In both growth conditions, LacZ activity in the complementation strain (Δ*cra* Δ*crp* P*_fruB_*-*lacZ* pJML05::*cra*) was significantly less than LacZ activity in the mutant strain containing an empty vector (Δ*cra* Δ*crp* P*_fruB_*-*lacZ* pJML05; *P < *0.05 by Tukey’s multiple-comparison test) (Fig. 6A). We conclude that Cra represses *fruB* expression by directly or indirectly affecting some portion of the *fruB* promoter included in the P*_fruB_* reporter.

The *crp* mutant strain, on the other hand, produced 5-fold lower LacZ activity (*P < *0.05, Tukey’s multiple-comparison test) in both fructose and nonfructose growth conditions compared to the “WT strain” containing the endogenous *crp* locus (Fig. 6B). Also in both growth conditions, LacZ activity in the complementation strain (Δ*cra* Δ*crp* P*_fruB_*-*lacZ* pTrc99A::*crp*) was 3-fold higher than the mutant strain containing an empty vector (Δ*cra* Δ*crp* P*_fruB_*-*lacZ* pTrc99A) (Fig. 6B), suggesting partial complementation. Overall, these results support a role for CRP in activating *fruB* transcription in both fructose and nonfructose conditions by acting, directly or indirectly, on some portion of the *fruB* promoter included in the P*_fruB_* reporter. Collectively, data presented in Fig. 6 also indicate that Cra and CRP can work independently of the other to regulate *fruB* expression.

### Determination of Cra and CRP regulatory sites.

**FIG 7:**
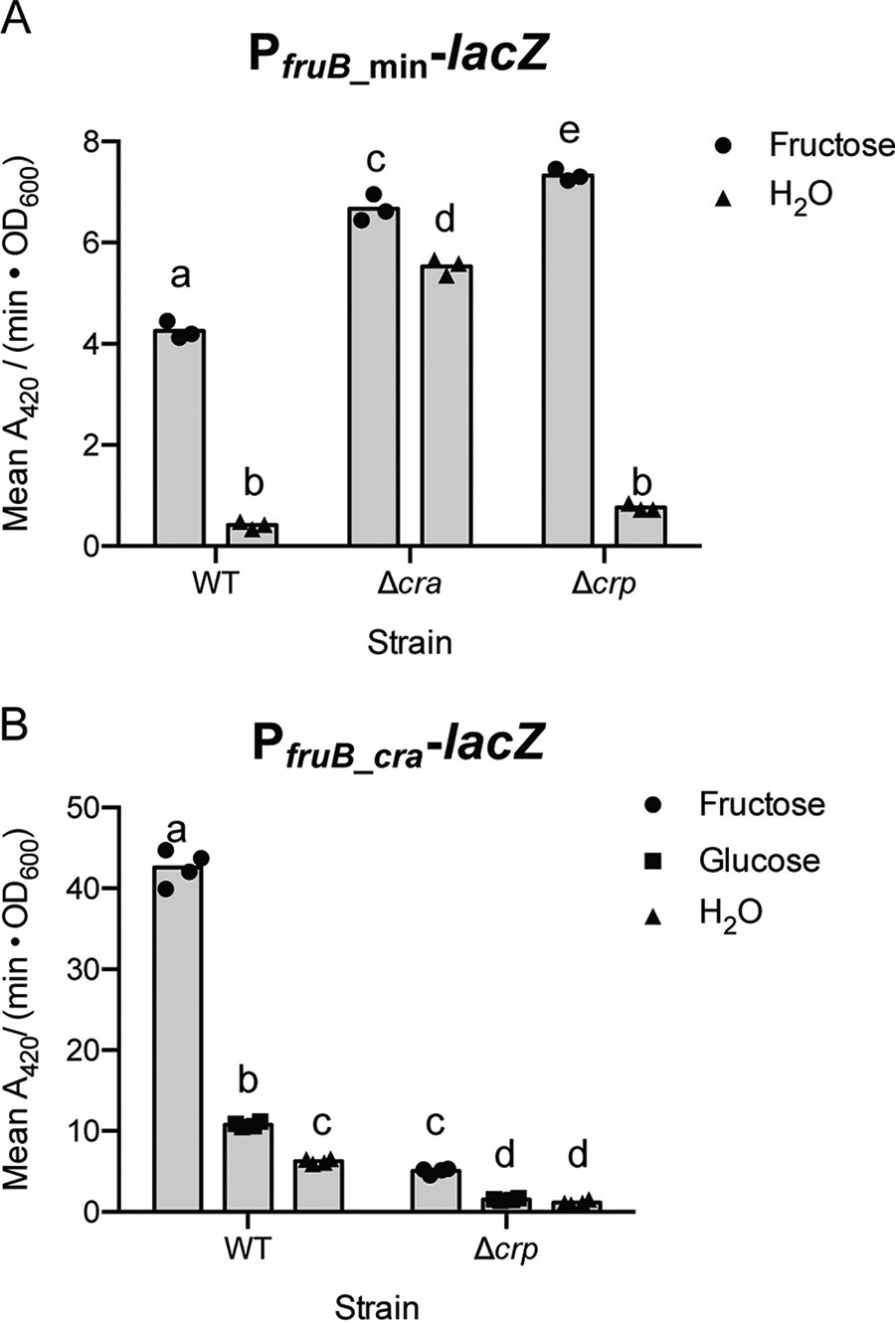
Cra acts near the predicted −10 and −35 sites of the *fruB* promoter, while CRP acts farther upstream. (A) *fruB* promoter activity from P*_fruB_*__min_ transcriptional reporter in wild-type, Δ*cra*, and Δ*crp* strains. (B) *fruB* promoter activity from P*_fruB_*__crp_ transcriptional reporter in wild-type and Δ*crp* strains. Strains were initially grown in LB and back diluted into fresh cultures supplemented with 0.4% (wt/vol) fructose, glucose, or an equivalent volume of water. After reaching an OD_600_ of 1.0, cultures were lysed and incubated with ONPG substrate solution for 1 h, from which *A*_420_/minute was measured and normalized to OD_600_. For each carbon source and strain, cultures were grown in biological replicates and measured in technical triplicate. Technical triplicates were averaged during data analysis. Bars represent means from biological replicates. Within each panel, bars with different letters indicate mean values that are significantly different from each other (*P < *0.05, Tukey’s multiple-comparison test).

Having established Cra and CRP as transcriptional regulators of *fruB* expression using the P*_fruB_* reporter, we then constructed additional transcriptional reporter fusions to identify regulatory sites for each regulator in the *fruB* promoter. In constructing these reporters, we considered the locations of putative regulatory sites for Cra and CRP to act directly on the promoter, based on computational predictions and their similarities to consensus sequences in E. coli and V. cholerae. BPROM identified only one putative Cra site starting at position −12 in relation to *fruB* TSS-1 (Fig. 2 and Fig. S1). Moreover, the sequence of this putative site, TGAATC-GATTCA, aligns well with the palindromic Cra consensus sequence previously identified in E. coli, TGAAAC-GTTTCA (20–23, 29). To evaluate the validity of this predicted regulatory site, we constructed the P*_fruB__*_min_ reporter, which spans −50 to +10 of the *fruB* promoter region and is smaller than the P*_fruB_* reporter (Fig. 2). We then measured LacZ activity from the P*_fruB__*_min_ reporter in WT and Δ*cra* strains in rich media supplemented with fructose or, as a control, water. We observed a 10-fold induction of LacZ activity when the P*_fruB_*__min_ reporter strain was grown in the presence of fructose, and this induction was reduced to 1.2-fold when *cra* was absent (*P < *0.05, Tukey’s multiple-comparison test) (Fig. 7A). These observations suggest that the promoter, as well as a TSS, are still intact within the P*_fruB__*_min_ reporter. In the *cra* mutant strain, LacZ activity from the P*_fruB__*_min_ reporter increased in both growth conditions compared to the WT strain (*P < *0.05, Tukey’s multiple-comparison test), indicating that Cra-mediated regulation is still taking place within the 60 nt of the *fruB* promoter included in the P*_fruB__*_min_ reporter and is responsible for the majority of the induction observed from this minimized promoter. This region includes the sequence that spans the predicted −10 and −35 hexamers, where a transcriptional repressor has the opportunity to enact a large effect on RNA polymerase recruitment or activity (30). We also note that the original P*_fruB_* reporter presented a 20- to 37-fold induction upon addition of fructose to the growth medium (Fig. 3A) compared to the 10-fold induction obtained using the P*_fruB_*__min_ construct. Thus, while the P*_fruB_*__min_-*lacZ* reporter is induced by fructose, P*_fruB__*_min_ may exclude regulatory sites for a transcriptional activator or an additional TSS needed for maximal induction.

We also evaluated the P*_fruB__*_min_ reporter in the context of CRP. LacZ activity from the P*_fruB__*_min_ reporter in a *crp* mutant strain presented 10-fold induction in the presence of fructose, similar to what was observed in the WT strain (*P < *0.05, Tukey’s multiple-comparison test) (Fig. 7A), which suggests that regulatory sites responsive to CRP are not included in the P*_fruB_*__min_ construct. We noted, however, that in fructose-supplemented medium, deletion of *crp* led to a 1.7-fold increase in LacZ activity compared to the WT (*P < *0.05, Tukey’s multiple-comparison test). This is inconsistent with CRP functioning as a transcriptional activator (Fig. 5B and 6B). Our transcriptional reporters are integrated, in an antisense direction, into the native *lacZ* gene in the V. cholerae genome, which is activated by CRP (data not shown). We postulate that the observed changes in LacZ activity when comparing P*_fruB_*__min_ in the WT and Δ*crp* strains in certain growth conditions may represent changes in antisense transcriptional readthrough that become more prominent given the low activity of the P*_fruB_*__min_ construct.

To account for promoter regions through which CRP activates *fruB* expression, we used the search tool Virtual Footprint, which predicted a CRP site lying 61 nt upstream of the *fruB* TSS (TSS-1) (Fig. 2 and Fig. S1) (31). The location of this predicted regulatory site almost perfectly matches the location of the CRP site known to exist in the E. coli *fruB* promoter, which lies 60 nt upstream of the *fruB* TSS (25). Additionally, the sequence of this putative site, TGTGC-GTCTGA-TCATA, is in good agreement with the sequence of the CRP box motif previously identified in E. coli, TGTGA-NNNNNN-TCACA (25).

To validate or invalidate this predicted site, we constructed the P*_fruB__*_crp_ reporter, which spans −93 to +10 of the *fruB* promoter and includes the predicted CRP box (Fig. 2). We then measured LacZ activity from this reporter in WT and Δ*crp* strains (Fig. 7B). Consistent with our assessment that CRP is an activator of *fruB*, across all culture conditions, LacZ activity from the P*_fruB__*_crp_ reporter decreased significantly in the *crp* mutant strain compared to the WT (*P < *0.05, Tukey’s multiple-comparison test). Also, even in the absence of *crp*, the addition of fructose to the growth medium induced LacZ activity 3- to 4-fold, compared to 4- to 7-fold in the WT (*P < *0.05, Tukey’s multiple-comparison test). This change in fold induction likely represents the loss of the activator, CRP, but maintenance of the repressor, Cra. Collectively, LacZ activity from both P*_fruB__*_min_ and P*_fruB__*_crp_ reporters allows us to narrow the location of a putative CRP binding site to a 43-nt region that lies within the region of *fruB* promoter included in the P*_fruB__*_crp_ reporter but excluded from the P*_fruB__*_min_ reporter.

### CRP represses *cra* expression.

**FIG 8:**
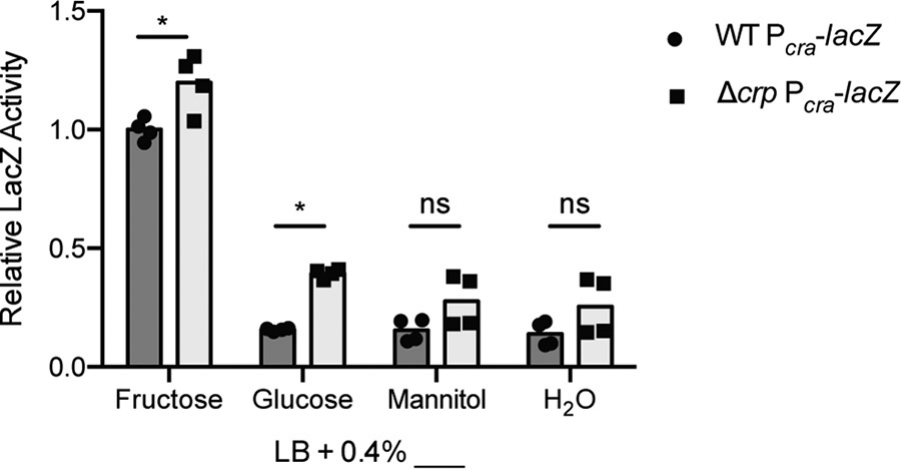
CRP represses *cra* expression in fructose and glucose growth conditions. *cra* promoter activity in multiple carbon sources in the presence and absence of CRP as measured by mean *A*_420_/minute from the P*_cra_-lacZ* transcriptional fusion. Strains were initially grown in LB overnight and back diluted into fresh cultures supplemented with 0.4% (wt/vol) fructose, glucose, mannitol, or an equivalent volume of water the following day. After reaching an OD_600_ of 1.0, cultures were lysed and incubated with ONPG substrate solution for 1 h, from which *A*_420_/minute was measured and normalized to OD_600_. A baseline value was obtained by averaging all replicates from the WT P*_cra-_lacZ* reporter in fructose conditions. Relative LacZ activity for other conditions and reporters was calculated by dividing the mean *A*_420_/(minute × OD_600_) value by the baseline, which was set to 1. For each carbon source, cultures were grown in biological replicate and measured in technical triplicate. Technical triplicates were averaged during data analysis. *, pairwise differences when comparing different strains in the same carbon source were significant at *P < *0.05; ns, not significant (Sidak’s multiple-comparison test).

In the above-described assays, we established that Cra and CRP affect *fruB* expression in the absence of the other regulator (Fig. 6). However, these assays fail to consider the possibility that the two regulators also interact in modulating *fruB* expression. The CRP binding site predicted by Virtual Footprint, which lies in the *fruB*-*cra* intergenic region, resides in close proximity to the *cra* TSS lying on the opposite strand (Fig. 2). To test whether the expression of *cra* is dependent on CRP, we constructed a transcriptional reporter containing the *cra* promoter region (Fig. S5 and S6). We then measured LacZ activity from the P*_cra_* reporter in WT and Δ*crp* strains in LB containing fructose, glucose, mannitol, or, as a negative control, water (Fig. 8). In both mannitol and negative-control conditions, no significant changes in LacZ activity between strains were observed (*P > *0.05, Sidak’s multiple-comparison test) (Fig. 8). In both fructose and glucose conditions, however, we observed significant increases in LacZ activity in the *crp* mutant strain compared to WT, which suggests that CRP may repress *cra* expression in the presence of these carbon sources. We postulate that in the presence of fructose, CRP prevents unnecessary, excess synthesis of Cra, and in glucose conditions, CRP may “rein” in Cra to allow for some basal levels of FPr to be synthesized such that the bacteria are primed to metabolize fructose once it is available.

## DISCUSSION

Important to the survival of V. cholerae is its ability to respond to changes in carbohydrate availability, depending on its current environment (7). The series of carbohydrate-specific PTSs encoded in the V. cholerae genome is believed to be crucial to this process; PTS components are thought to be selectively produced in order to transport and phosphorylate available carbon sources into the bacteria for metabolism (7, 10, 11). Here, we describe the transcriptional regulation of *fruB*, which encodes the FPr component of PTS^Fru^, in an effort to further dissect how V. cholerae responds to changes in carbohydrate availability. We show that *fruB* is expressed at the highest levels in fructose media, which is consistent with data collected during a similar study in Pseudomonas putida, the only other Gram-negative bacterium that, to our knowledge, has been used to study *fruB* using a transcriptional reporter system (32). Expression of *fruB* is controlled by both Cra and CRP, which impact transcription through different regions of the *fruB* promoter in response to the absence and presence of fructose. The proximal location of *fruK* and *fruA* to *fruB*, and their parallel induction by fructose, further suggests that the three genes may be coregulated by Cra and CRP.

Using the P*_fruB_* reporter, we showed that the *fruB* TSS lies approximately 241 nt upstream of its start codon, in line with previously published dRNA-Seq data from Papenfort and colleagues (Fig. 2 and 3; see Fig. S1 in the supplemental material) (16). This conflicts with the TSS we determined by 5′ RACE, which lies 133 nt upstream of the *fruB* start codon (Fig. 2; Fig. S1 and S2). Interestingly, the intergenic transcript IGR4 (107 nt) lies directly between these two sites, spanning almost the entire length between the two markers (Fig. S1). IGR4 was first identified in a massively parallel sequencing experiment which aimed to uncover novel small RNAs (sRNAs) with regulatory roles in V. cholerae (33). Because of its proximity to the *cra* promoter region, IGR4 was hypothesized to be a putative *cis*-acting sRNA, imparting regulation through extensive binding to the *cra* promoter (34). However, Western blotting probing for Cra in a strain overexpressing IGR4 suggests the small transcript has no regulatory effect on Cra levels (Fig. S7). One possible explanation for the range of 5′ ends observed in our RNA-Seq data, our aberrant 5′ RACE results, as well as the existence of IGR4, is the occurrence of posttranscriptional processing of the *fruB* transcript by an endonuclease. RNase E, arguably the most prominent RNase in E. coli, and YbeY are both promising candidates for cleavage of *fruB* mRNA; these two endonucleases have also been shown to target transcripts in V. cholerae (35–38).

Using P*_fruB_*__min_ and P*_fruB_*__crp_ reporters, we identified sites through which Cra and CRP influence *fruB* transcription, possibly through direct interactions with the *fruB* promoter; the likely locations of −10 and −35 hexamers of the *fruB* promoter were also mapped (Fig. 7A and B). The sequences of predicted regulator binding sites identified here agree well with previously determined consensus sequences from E. coli (Fig. S1) (20–23, 25, 29). In the V. cholerae genome, the −10 hexamer shows a distinct consensus sequence, TAnaaT (spanning −12 to −7 sites), and highly conserved bases at positions −11 and −7 (adenine and thymine, respectively) are maintained in the sequence of the −10 hexamer we predict here, CAGTAT (16). While the manuscript was under review, a separate study was published that demonstrated the binding of Cra to the putative Cra binding site we included in the P*_fruB_*__min_-*lacZ* construct (Fig. 2 and Fig. S1) (39). Interestingly, the authors propose that in some growth conditions, Cra may recruit RNA polymerase to the *fruB* promoter (39). These results, combined with those presented in this work, open up intriguing possibilities for further interrogation of the nuances of *fruB* expression throughout the V. cholerae life cycle.

In E. coli, Cra is frequently described as a pleiotropic regulator, involved in modulating the expression of a large number of genes, including *mtlA*, which encodes the EII^Mtl^ protein (22). While Cra-mediated repression of the *fru* operon in V. cholerae closely mimics the same process in E. coli, it is unclear if a similar Cra regulon exists in V. cholerae. Western blotting probing for the PTS^Mtl^ transporter MtlA in WT and Δ*cra* strains suggests the V. cholerae Cra regulon may not include the same targets as in E. coli; MtlA levels did not vary between WT and mutant strains in V. cholerae, although Cra has been shown to repress MtlA in E. coli (20) (Fig. S8). Our RNA-Seq data further confirm this finding. Thus, although Cra regulates *fruB* expression in both E. coli and V. cholerae, their regulons likely contain divergent targets.

One peculiarity in our description of Cra’s activity in the *fruB* promoter is the repressor’s individual expression pattern. LacZ activity from the P*_cra_* reporter and analogous Western blotting probing for Cra indicate that *cra* is expressed at the highest levels in fructose media, just like *fruB* (Fig. 8 and Fig. S8), and in the absence of fructose, Cra levels are low. This particular expression pattern is not intuitive given our observations of Cra activity. In fructose media, Cra is expressed at the highest levels when its activity as a repressor is expected to be minimal. In nonfructose media, Cra is expressed at much lower levels but is active in repressing *fruB*. Similar patterns of expression and activity have been observed in V. cholerae for the transcriptional regulator MtlR, which represses the genes encoding PTS^Mtl^ in the absence of the sugar alcohol mannitol (40). In the case of Cra, fructose-1-phosphate (F1P) may provide an explanation for these unexpected activity patterns. We propose a model that when fructose is imported into the cell, it is first phosphorylated to F1P as it crosses the inner bacterial membrane and is then phosphorylated again once inside by 1-phosphofructokinase (the protein product of *fruK*) to become fructose-1,6-bisphosphate (FBP) (12). In E. coli, F1P has been shown to bind Cra and significantly inhibit Cra’s DNA binding ability (41). Considering the similarities between E. coli *cra* and its V. cholerae homolog, we hypothesize F1P likely plays a similar role in V. cholerae and could help to explain why Cra is inactive in fructose media, even when expressed at relatively high levels. The enzymatic activity of FruK, which converts F1P to FBP, may initiate a feedback loop that controls Cra activity; FruK’s activity essentially removes the effector molecule (i.e., F1P) which dampens Cra activity. When the concentration of F1P decreases, Cra becomes active and represses *fruB* transcription, which may, in turn, also decrease expression of *fruK*, consequently increasing the concentration of F1P. As this concentration increases, Cra becomes inactive, *fruB* expression increases, and the cycle repeats itself.

## MATERIALS AND METHODS

### Strains and culture conditions.

All strains used in this study are described in Table S1 in the supplemental material. The wild-type V. cholerae strain used in this study, from which all subsequent strains were constructed, was the O1 biovar El Tor N16961 Δ*tcpA* strain. The *tcpA* mutant is highly attenuated for virulence and was used for safety purposes. Unless otherwise denoted, “wild type” refers to the N16961 Δ*tcpA* strain.

All strains were streaked onto Luria-Bertani (LB) plates with the appropriate antibiotics and incubated at 37°C for 12 to 16 h. Liquid cultures were prepared by inoculating 2 ml LB broth or 1× M9 minimal medium containing one or more carbon sources (totaling 0.4% [wt/vol]) with individual colonies. All cultures were supplemented with the appropriate antibiotics at the following concentrations: streptomycin (Sm) at 100 μg/ml and carbenicillin (Cb) at 50 to 100 μg/ml. Cultures prepared in 1× M9 minimal medium were also supplemented with 0.1% (wt/vol) trace metals (5% MgSO_4_, 0.5% MnCl_2_, 0.5% FeCl_3_, and 0.4% nitrilotriacetic acid). Unless otherwise stated, cultures were incubated at 37°C for 12 to 16 h, with shaking at 250 rpm.

### Mutant strain construction.

V. cholerae strains harboring chromosomal mutations were constructed as follows. A plasmid bearing the desired mutation was constructed in the allelic exchange vector pCVD442 via splicing by overlap extension (SOE) PCR. Two roughly 600-bp DNA fragments flanking the region of interest were amplified by PCR using F1/R1 and F2/R2 primer pairs (Table S2). These fragments were annealed together and then amplified by PCR using the F1 and R2 primers. The final PCR product was assembled via Hi-Fi DNA assembly (New England BioLabs) with the pCVD442 backbone, which was prepared using the appropriate pCVD_F and pCVD_R primers (Table S2). The resultant plasmid was propagated in E. coli DH5α*λ*pir and then transformed into E. coli SM10*λ*pir before being conjugated into V. cholerae. Successful conjugates were selected from one round of growth in LB broth with streptomycin, and the resultant colonies were plated on sucrose medium to screen for successful vector disintegration. Sucrose-resistant colonies were screened for the desired mutation by PCR with the F0 and R0 primers and confirmed by sequencing (Eurofins).

### Transcriptional reporter construction.

To assemble the *lacZ* transcriptional fusion reporters, we generated a series of derivatives of the pJL1::*lacZ*(*Ec*) plasmid, which contains the ribosome binding site (RBS) and coding sequence of E. coli *lacZ* [*lacZ*(*Ec*)] inserted into a fragment of the VC2338 locus (encoding the V. cholerae homolog of *lacZ*) in the antisense orientation (42). In each derivative plasmid, we inserted the desired portion of the *fruB* promoter directly upstream of the *lacZ*(*Ec*) RBS. To construct derivative plasmids for P*_fruB_* and P*_fruB_*__min_ reporters, DNA fragments containing the desired portion of the *fruB* promoter were amplified by PCR using forward insert and reverse insert primer pairs (Table S2). The derivative plasmid was then assembled via Hi-Fi DNA assembly (New England BioLabs) with the amplified DNA fragment and the pJL1::*lacZ*(*Ec*) backbone, which was amplified by PCR using forward vector and reverse vector primers (Table S2). To construct derivative plasmids for P*_fruB_*__crp_ and P*_fruB_*__null_ reporters, desired double-stranded DNA (dsDNA) fragments of the *fruB* promoter (gBlocks) were ordered from Integrated DNA Technologies, and plasmids were assembled via Hi-Fi DNA assembly as described for P*_fruB_* and P*_fruB_*__min_ reporters. The exact coordinates of the *fruB* promoter region included in each fusion are indicated in Table S1.

Derivative plasmids were then propagated in E. coli DH5α*λ*pir, and correct assembly of the plasmid was checked by sequencing using LIU126 and LIU127 primers, which flank the site at which regions of the *fruB* promoter were inserted into pJL1::*lacZ*(*Ec*) (Table S2). Plasmids were then transformed into E. coli SM10*λ*pir before being conjugated into V. cholerae, using the sucrose-screening method described above.

### Complementation plasmid construction.

All plasmids were constructed using the Hi-Fi master mix (New England BioLabs) to assemble DNA fragments. Plasmid pTrc99a::*crp* was obtained using PCR fragments amplified using primers LIU152-153 (to amplify the pTrc99a backbone) and LIU154-155 (to amplify *crp* from the V. cholerae genome). Vector pJML05 was created by replacing the Ptrc promoter in the pTrc99a backbone with the PLlacO-1 promoter. This was accomplished using primers LIU476-477 to amplify the backbone of pTrc99a and DNA oligonucleotide LIU480. Plasmid pJML05::*cra* was assembled using PCR products derived by using primers LIU652-653 (to amplify the backbone of pJML05) and LIU654-655 (to amplify *cra* from the V. cholerae genome). Plasmid pJML05::IGR4 was assembled from PCR products obtained using primers LIU494-495 (to amplify the backbone of pJML05) and LIU496-497 (to amplify the IGR4 sequence from the V. cholerae genome). All constructs were confirmed by sequencing.

### RNA-Seq experiments and analysis.

For each combination of strain and growth sample, RNA samples in biological duplicate were prepared. Cells from LB agar plates were used to inoculate 2 ml of 1× M9 minimal medium supplemented with 0.4% glucose or fructose (wt/vol). Overnight cultures were diluted into 2 ml fresh 1× M9 medium with 0.4% glucose or fructose (starting optical density at 600 nm [OD_600_], ∼0.05) and grown to an OD_600_ of ∼0.3. The entire culture was harvested (8,000 × *g*, 5 min, 4°C), and the RNA was purified from the cells using the Direct-zol RNA miniprep kit (Zymo Research) following the manufacturer’s instructions. Eluted samples were treated with DNase at 37°C for 30 min, twice, using Turbo DNA-free (Ambion) according to the manufacturer’s protocol. RNA integrity was initially analyzed by agarose gel electrophoresis. Additional RNA integrity analysis, rRNA depletion, cDNA synthesis, and library preparation and sequencing were performed by Quick Biology (Pasadena, CA). Paired-end, 150-bp sequences were generated for 10 million reads per sample.

Fastq files were mapped to the V. cholerae biovar El Tor strain N16961 genome (NCBI accession no. GCA_000006745.1) using the BWA-MEM algorithm in BWA version 0.7.12 (43). Aligned reads were counted with htseq-count version 0.11.2 (44) with the intersection-strict argument. Counts were imported into R version 3.6.2 (45), and differential expression was assessed with DEseq2 version 1.26.0 (46).

### General total RNA extraction.

RNA for 5′ RACE and qRT-PCR was extracted from overnight V. cholerae cultures that were back diluted and grown to mid-log phase (OD_600_, ∼0.3). Total RNA was extracted using the Direct-zol RNA miniprep kit (Zymo) following the manufacturer’s instructions. For qRT-PCR, eluted samples were treated with DNase at 37°C for 30 min using Turbo DNA-free (Ambion) according to the manufacturer’s protocol.

### qRT-PCR.

Determination of relative expression levels was performed on total RNA using the Stratagene MX3005P system, the Brilliant II SYBR green qRT-PCR master mix kit (Agilent), and primers specific to *fruB*, *fruK*, *fruA*, and 4.5S (Table S2 in the supplemental material). The reactions were set up in 96-well optical reaction plates and contained 1× Brilliant SYBR green qPCR master mix, 30 nM ROX reference dye, each primer at 100 nM, 100 ng RNA, and 1 μl RT/RNase block enzyme mixture in a 25-μl reaction mixture. The following conditions were used for cDNA synthesis and PCR: 30 min at 50°C, 10 min at 95°C, and 40 cycles of 30 s at 95°C and 1 min at 60°C (Agilent). MxPro QPCR software (v. 4.10) was used to determine threshold cycle (*C_T_*) values for each reaction, and relative RNA concentrations were calculated from the *C_T_* values by comparison to standard curves. All transcript levels were normalized to a 4.5S RNA endogenous control. No signals were detected in no-template controls and no-reverse transcriptase (RT) controls.

### 5′ RACE.

5′ rapid amplification of cDNA ends (RACE; Invitrogen) was performed according to the manufacturer’s instructions. Gene-specific primers (GSPs) (Table S2) were designed to anneal to the coding region of the gene of interest (i.e., *cra* or *fruB*) in order to synthesize cDNA and amplify the upstream region from extracted RNA samples. Amplified PCR products were introduced into the pCR4-TOPO vector using the TOPO TA cloning kit (Invitrogen). Plasmids were then isolated and sequenced using M13 forward and M13 reverse primers (Eurofins).

### Transcriptional reporter assays.

All transcriptional reporter assays were performed using strains containing a *lacZ*(*Ec*) gene construct that was inserted into the endogenous *lacZ* gene in order to disrupt native *lacZ* expression. Bacterial samples were taken from liquid cultures which were back diluted and grown to late log phase (OD_600_, ∼1.0). In strains harboring the pJML05 plasmid or a derivative, 5 mM isopropyl-β-D-thiogalactopyranoside (IPTG) was added to back dilutions to induce expression from the PLlacO-1 promoter. In strains harboring the pTrc99a plasmid or a derivative, 1 mM IPTG was added to back dilutions to induce expression from the *trc* promoter. Cell samples (200 μl) were loaded into a clear 96-well plate in replicate, and OD_600_ measurements were taken using a Synergy 4 plate reader (BioTek). From these samples, 100 μl of cells were lysed for 25 to 35 min with a 10-μl solution containing PopCulture reagent (Novagen) and lysozyme (Thermo Fisher) in a 1,000:1 ratio. Samples (30 μl) of cell lysate were then incubated with 150 μl of *o*-nitrophenyl-β-d-galactopyranoside (ONPG) substrate solution (60 mM Na_2_HPO_4_, 40 mM NaH_2_PO_4_, 1 mg/ml ONPG, and 2.7 μl/ml β-mercaptoethanol) in a separate 96-well plate at 28°C. The absorbance at 420 nm (OD_420_) was recorded every 30 s over 60 min by a Synergy 4 plate reader (BioTek). Final results (denoted in the text as LacZ activity) are reported as the average slope (in mean OD_420_ per minute) of the 30-s intervals over the course of the 60-minute incubation period normalized to OD_600_ (mean OD_420_/[minute × OD_600_]). Technical replicates were averaged during data analysis and statistical analyses were performed using GraphPad Prism (version 7) software.

### Western blotting.

For FPr-FLAG and MtlA-FLAG analysis, cell pellets were prepared from 2 ml back-diluted cultures grown to mid-log phase (OD_600_, ∼0.3). Following centrifugation at 8,000 × *g* for 5 min at 4°C, pellets were resuspended in LB or 1× M9 medium, mixed 1:4 in SDS sample buffer (250 mM Tris-HCl [pH 6.8], 10% SDS, 50% glycerol, 10% β-mercaptoethanol, and 0.5% orange G), and heated at 95°C for 10 min. Samples were loaded onto an SDS containing 4 to 20% Tris gel (Bio-Rad) and run at 200 V for 25 min. Proteins were then transferred to a nitrocellulose membrane using a Trans-Blot Turbo transfer system (7 min at 1.3 A; Bio-Rad). Revert total protein stain (Li-Cor), a near-infrared fluorescent membrane stain, was then used to stain all protein on the membrane following the manufacturer’s instructions. Revert stain was detected at 700 nm using an Odyssey imager (Li-Cor). The membrane was then incubated with a dilution of primary antibody (1:5,000 of rabbit anti-FLAG [Abcam]) for 1 h, followed by incubation with a dilution of secondary antibody (1:10,000 of IR800-conjugated goat anti-rabbit immunoglobulin [Li-Cor]) for 30 min. Signal was visualized at 800 nm using an Odyssey imager (Li-Cor), and ImageStudio software (version 5; Li-Cor) was used to quantify fluorescent signal and normalize values to Revert total protein measurements.

For Cra-hemagglutinin (HA) analysis, cell pellets were prepared from 50-ml back-diluted cultures grown to mid-log phase (OD_600_, ∼0.3). Pellets were lysed with B-PER bacterial protein extraction reagent (Thermo Scientific) in the presence of DNase I (Thermo Scientific) following the manufacturer’s instructions. Following protein extraction, the same methods used for FruB-FLAG and MtlA-FLAG analysis (described above) were used. During the immunodetection steps, membranes were incubated with rabbit anti-HA antibody (Abcam) in a 1:1,000 dilution for 1 h, followed by a 30-minute incubation with IR800-conjugated goat anti-rabbit (Li-Cor) antibody in a 1:6,667 dilution.

### Data availability.

RNA-Seq data were deposited in the GEO database with accession number GSE164298.

## Supplementary Material

Supplemental file 1
