# Supplementary material for: Cra and cAMP Receptor Protein Have Opposing Roles in the Regulation of fruB in Vibrio cholerae
Source: J Bacteriol. 2021 Apr 21;203(10):e00044-21. doi: 10.1128/JB.00044-21 (PMC8088597; doi:10.1128/JB.00044-21)
Supplement: Supplemental file 1 [file JB.00044-21-s0001.pdf]

Cra and CRP Have Opposing Roles in the Regulation of the *fruB* in *Vibrio cholerae*

Christina Beck,[a]* Sayde Perry,[a] Daniel M. Stoebel,[b] and Jane M. Liu[a]#

[a]Department of Chemistry, Pomona College, Claremont, California, USA
[b]Department of Biology, Harvey Mudd College, Claremont, California, USA

#Address correspondence to Jane M. Liu, jane.liu@pomona.edu.
*Present address: Christina Beck, Department of Biology, Massachusetts Institute of Technology, Cambridge, Massachusetts, USA

Supplemental Materials

Figure S1
Figure S2
Figure S3
Figure S4
Figure S5
Figure S6
Figure S7
Figure S8
Table S1
Table S2
References

```
                                                    CRP binding site
-113 TTTCATCCAGTGTCATAAGGGGGTCTCGTTTTATGTGCGTCTGATCATAGAACGCGATTTTTGAT

             cra TSS                       Cra binding site          fruB TSS-1
-48  CCTAGCCTAGTGTTGAATTATACGCTGAATCGATTCAGTATTAAAGCTGAAAGGATTCAGCAAAA
                   -35                               -10
                         IGR4
+18  GTACCGTTGATTCACAATCTCGTCCACTACAAAGGTCAGATTGTGTCGAGTATCCAGCAGCAAGA

                                  fruB TSS-2
+83  TCGTGAGTATTCGAGGTTTTGCTGAATTTTTTTGCCCTTTGTGCTGAACCGATTCAGCTCAAAGG

+148 AAAACGAGACGGGATGAATGACAAGGTATTGCTTCAACTGAAAACCTGCAGCGATACATCACATC

+213 TCGCCTATAGAGGCAGACAGGAGTTAAGAATGTTAGAACTCACTACACAAGATATTCAATTGCAG

+278 CAACACTTTGCGAATAAGCAAGCTGCGATTCAAGGACTGGCTCACGCGTTGACCGCGAAAGGCTT

+343 AGTGGCAGAAGGCTATGCGCAAGGTATGCTCAATCGCGAAGCACAGCATTCCACCTATCTCGGTA

+408 ATGGGATTGCGATTCCGCATGGCACAACCGATACCCGTGAACTGGTTAAACAGACAGGTGTCACC
                             fruB_5'RACE_GSP2
+473 GCCATGCACTTCCCGCAAGGTTTGGATTGGGGAGACGGTAATCTGGTGTATGTGGCCATCGGTAT
                                                        fruB_5'RACE_GSP1
+538 CGCCGCAAAATCGGATGAACATTTGGGCATTCTAAAGCAGCTGACCAGAGTGCTGTCTGCCGATG
```

Figure S1. Experimental *fruB* TSS differs from TSS from published RNA-Seq. Sequence of the antisense strand of the *fruB-cra* intergenic region, written 5' to 3'. Coding regions of *fruB* and *cra* are written in blue and green bolded text respectively. 5' RACE was used to determine the transcription start site of *fruB* (*fruB* TSS-2; written in blue and underlined) to be 133 nt upstream of the start codon (which is at position +242, relative to TSS-1). fruB_5'RACE_GSP1 and fruB_5'RACE_GSP2 reverse primers were used to reverse-transcribe transcripts in 5' RACE experiments. RNA used for 5' RACE was extracted from *V. cholerae* cultured in 1X M9 + 0.4% wt/vol fructose. This TSS differs from that identified by Papenfort et al., 2015 (*fruB* TSS-1; written in blue and underlined), which lies 241 nt upstream of the *fruB* start codon (1). The *cra* TSS (which lies on the sense strand) is written in green and underlined. Putative Cra and CRP binding sites are written in purple and red bolded text respectively. Putative *fruB* -10 and -35 hexamers are underlined. IGR4 (107 nt), a putative product of transcriptional processing, is highlighted in teal. Coordinates relative to the *fruB* TSS-1 +1 site are included on the left side of the sequence.

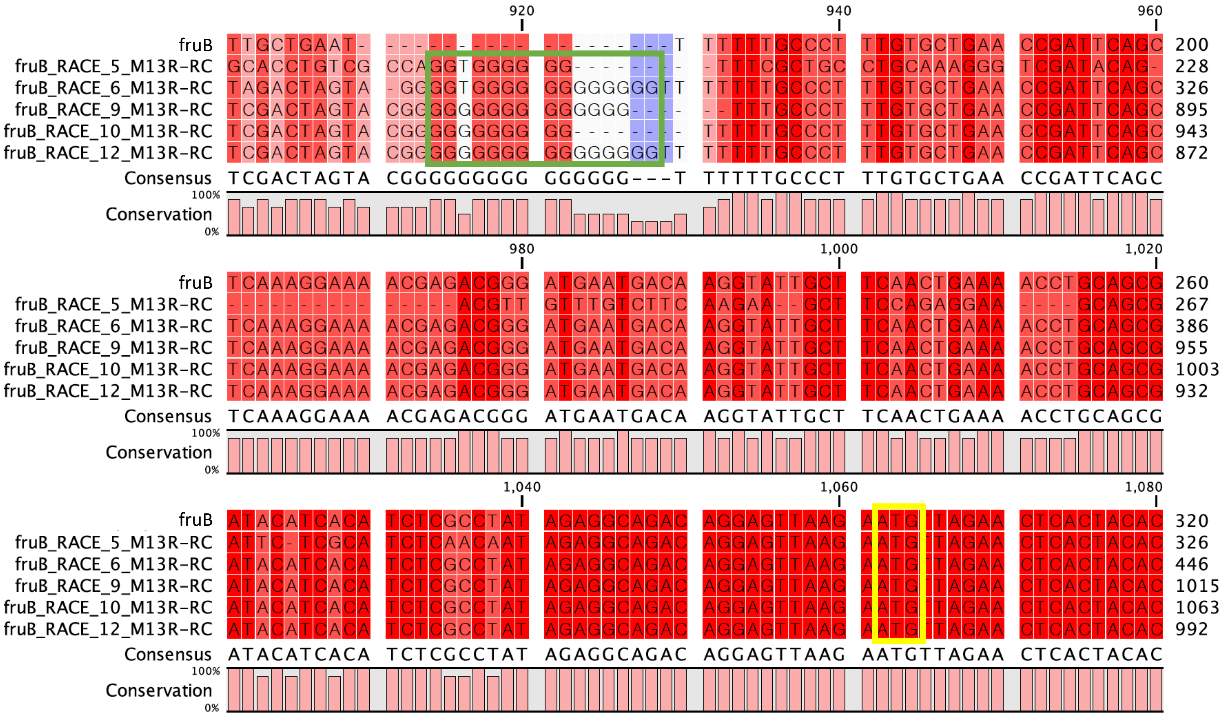

Figure S2. Consensus between *fruB* 5' RACE samples extracted from fructose cultures and sequence upstream of *fruB*'s start codon. The TSS is expected to lie directly downstream of the poly-dG tail synthesized during 5' RACE, which is highlighted by the green box. *fruB*'s start codon is highlighted by the yellow box. RNA for 5' RACE was extracted from *V. cholerae* cultured in 1X M9 + 0.4% wt/vol fructose, and fruB_5'RACE_GSP1 and fruB_5'RACE_GSP2 were used as primers in reverse transcription reactions. Amplified fragments were sequenced and aligned using CLC Sequence Viewer 7. 24 total sequences were analyzed across two separate experiments, and five sequences suggest that the TSS lies approximately 133 nt upstream of *fruB*'s start codon.

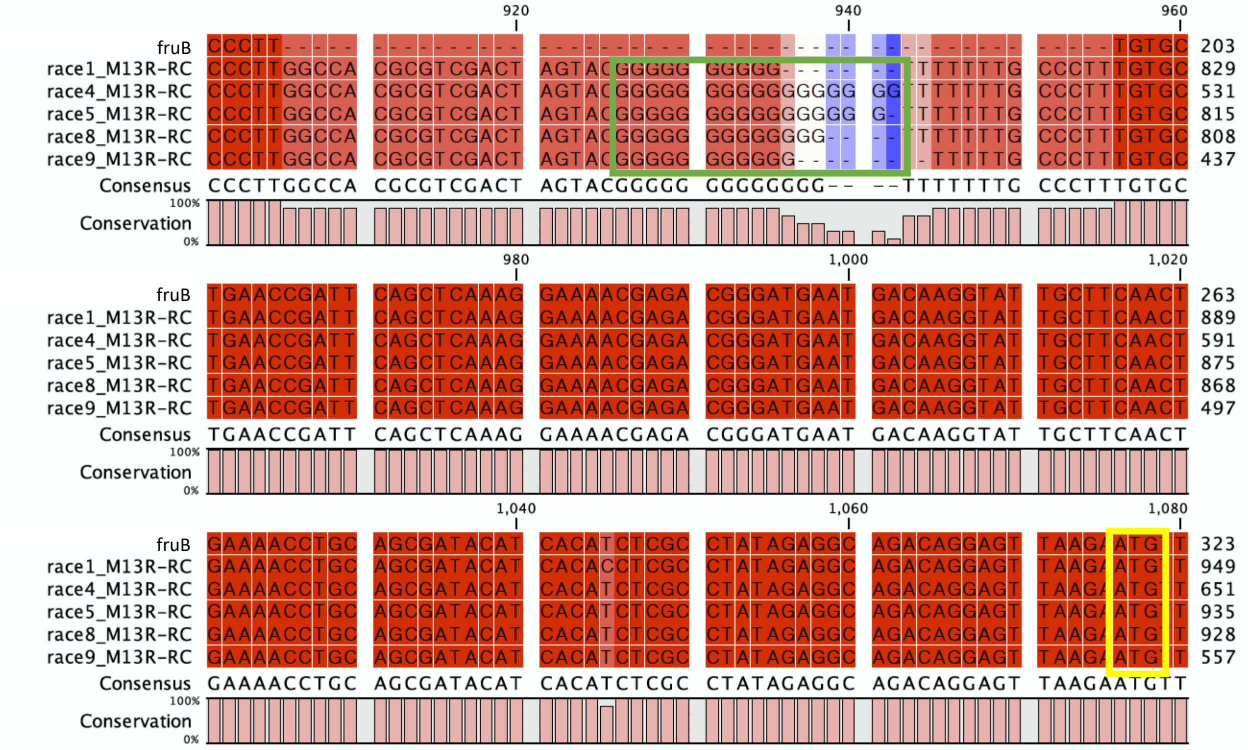

Figure S3. Consensus between *fruB* 5' RACE samples extracted from glucose cultures and sequence upstream of *fruB*'s start codon. The TSS is expected to lie directly downstream of the poly-dG tail synthesized during 5' RACE, which is highlighted by the green box. *fruB*'s start codon is highlighted by the yellow box. RNA for 5' RACE was extracted from *V. cholerae* cultured in 1X M9 + 0.4% wt/vol glucose, and fruB_5'RACE_GSP1 and fruB_5'RACE_GSP2 were used as primers in reverse transcription reactions. Amplified fragments were sequenced and aligned using CLC Sequence Viewer 7. 12 sequences were analyzed, and five sequences suggest that the TSS lies approximately 133 nt upstream of *fruB*'s start codon.

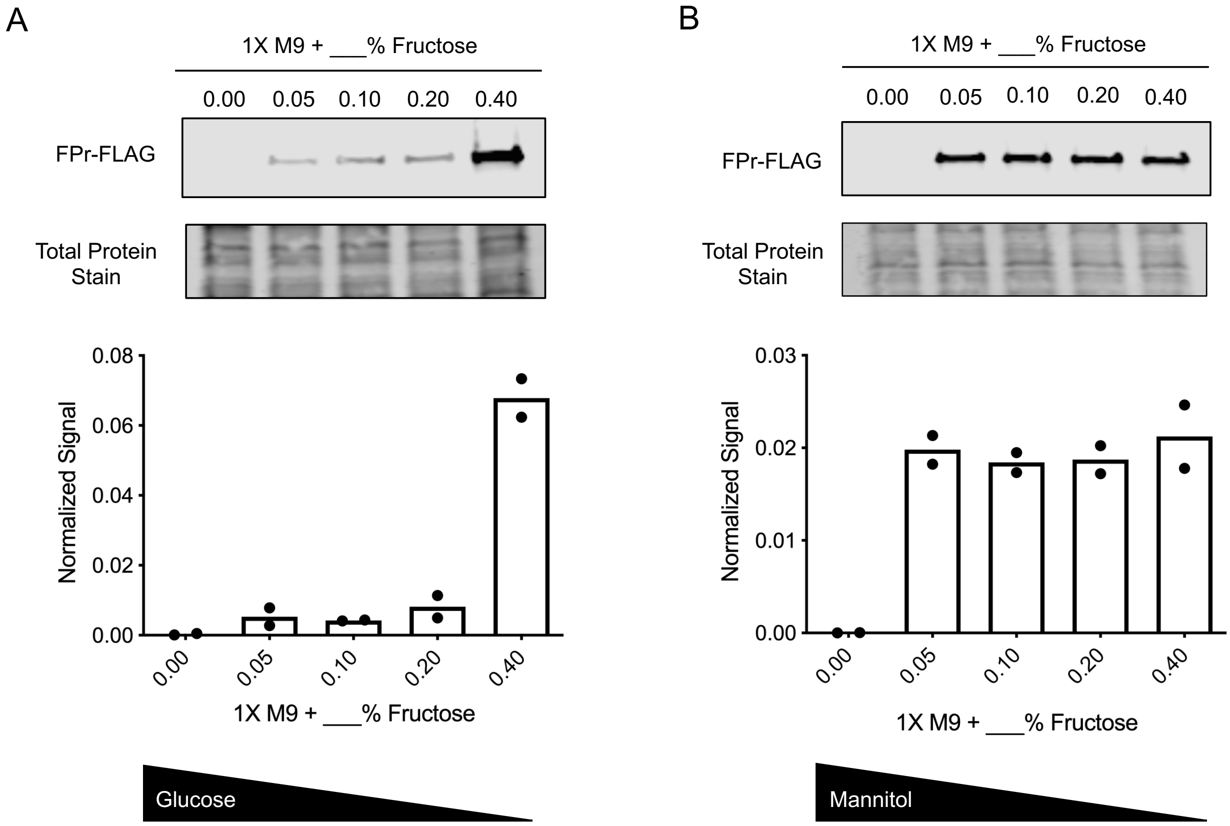

Figure S4. FPr levels are highest in fructose media. Western blots of FPr-FLAG (JL436) in 1X M9 supplemented with fructose and (**A**) glucose or (**B**) mannitol. Cultures were grown overnight in 1X M9 with the indicated mixtures of fructose and glucose or mannitol, totaling 0.4% wt/vol. The following day, cultures were back-diluted into fresh 1X M9 with the same mixtures of carbon sources as before. Back-dilutions were grown to mid log phase before protein extraction. 5 µL of the 100 µL protein extraction was included in loaded samples. Rabbit α-FLAG antibodies were used in Western blot analysis. Protein levels were normalized to REVERT Total Protein Stain.

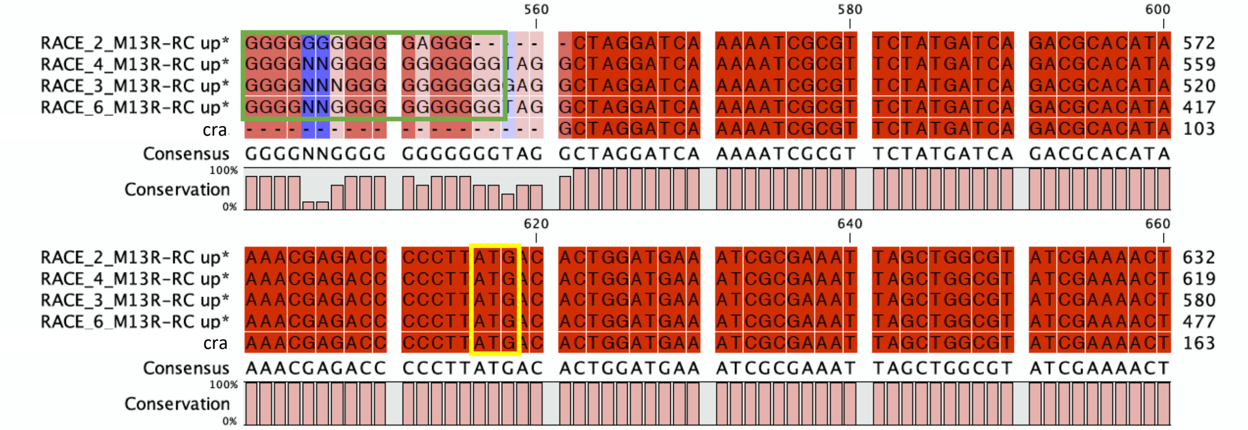

Figure S5. Consensus sequence between *cra* 5’ RACE samples and sequence upstream of *cra*’s start codon. The TSS is expected to lie directly downstream of the poly-dG tail synthesized during 5’ RACE, which is highlighted by the green box. *cra*’s start codon is highlighted by the yellow box. RNA for 5’ RACE was extracted from *V. cholerae* cultured in 1X M9 + 0.4% wt/vol fructose, and cra_5’RACE_GSP1 and cra_5’RACE_GSP2 were used as primers in reverse transcription reactions. Seven sequences were analyzed, and four sequences suggest that the TSS lies approximately 57 bp upstream of *cra*’s start codon.

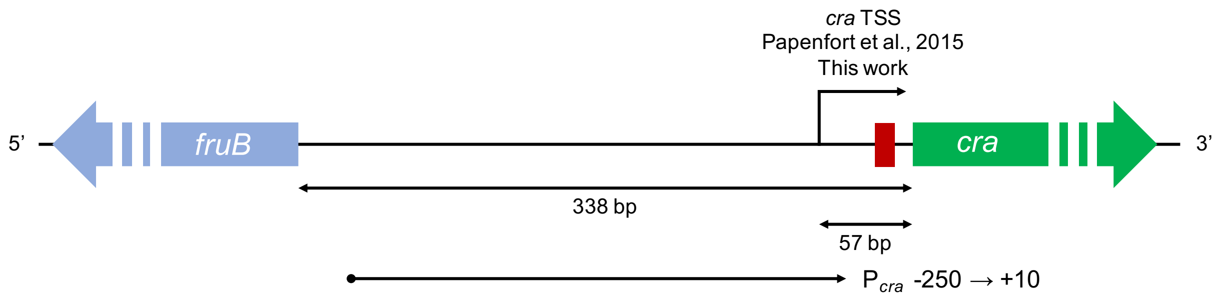

Figure S6. Schematic of P_{cra} transcriptional reporter design. The TSS of *cra* is indicated by the sideways arrow. This TSS was first determined by Papenfort et al., 2015 using RNA-Seq, and we observed the same TSS in this work using 5' RACE (RNA for 5' RACE was extracted from cultures grown in fructose media). The region of the *cra* promoter included in P_{cra} is indicated by the single-headed arrow. Exact coordinates for this region are listed to the right of the arrow, with numbering based on the *cra* TSS as +1. The red bar depicts a putative CRP binding site, which lies 14 nts upstream of *cra*'s start codon.

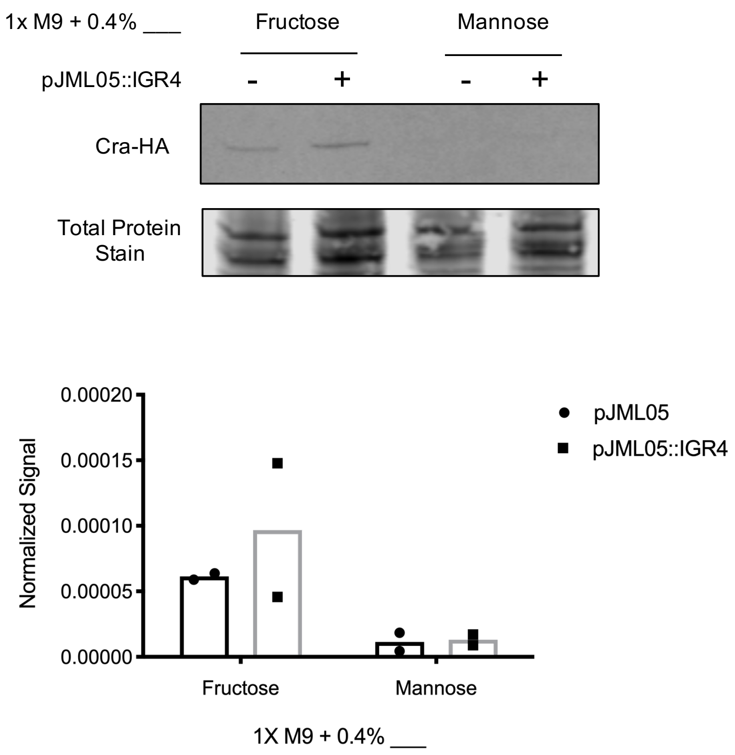

Figure S7. Cra levels remain unchanged when IGR4 is overexpressed. Western blot of Cra-HA pJML05 (JL530) and Cra-HA pJML05::IGR4 (JL531) in 1X M9 plus 0.4% wt/vol fructose or mannose. Protein was extracted using BPER. Rabbit α-HA antibodies were used in Western blot analysis. Protein levels were normalized to REVERT Total Protein Stain. The blots shown represent one of two experiments that are both included in the bar graph.

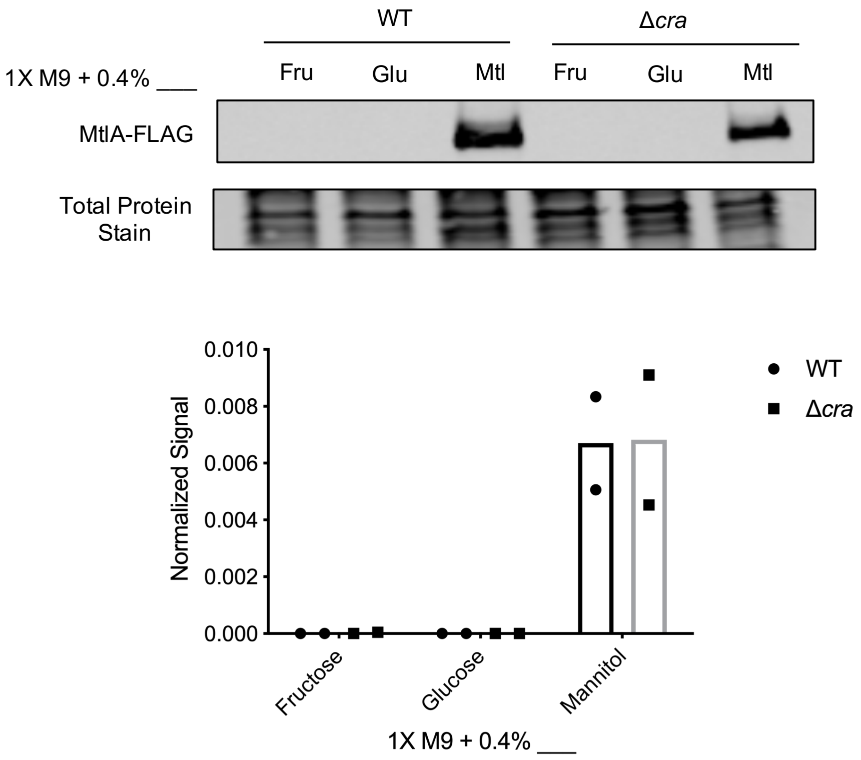

Figure S8. MtlA expression in the absence of Cra. Western blot of MtlA-FLAG (JL2) and Δ*cra* MtlA-FLAG (JL538) in 1X M9 plus 0.4% fructose, glucose, or mannitol. Rabbit α-FLAG antibodies were used in Western blot analysis. Protein levels were normalized to REVERT Total Protein Stain. The blots shown represent one of two experiments that are both included in the bar graph.

Table S1 Strains and plasmids used in this study

Strain or plasmid	Description or genotype[a]	Ref or Source
Strains		
V. cholerae		
JL1	N16961 Δ*tcpA* Sm^R	(2)
JL2	N16961 Δ*tcpA mtlA*-FLAG Sm^R	(2)
JL435	N16961 Δ*tcpA cra*-HA Sm^R	This study
JL436	N16961 Δ*tcpA fruB*-FLAG Sm^R	This study
JL461	N16961 Δ*tcpA* Δ*cra* Sm^R	This study
JL521	N16961 Δ*tcpA* Δ*cra fruB*-FLAG Sm^R	This study
JL530	N16961 Δ*tcpA cra*-HA pJML05 Sm^R Ap^R	This study
JL531	N16961 Δ*tcpA cra*-HA pJML05::IGR4 Sm^R Ap^R	This study
JL538	N16961 Δ*tcpA* Δ*cra mtlA*-FLAG Sm^R	This study
JL539	N16961 Δ*tcpA* Δ*crp fruB*-FLAG Sm^R	This study
JL558	N16961 Δ*tcpA* Δ*crp* Δ*cra fruB*-FLAG Sm^R	This study
JL567	N16961 Δ*tcpA fruB*-FLAG P_{fruB}-*lacZ* (-259 → +102) Sm^R	This study
JL568	N16961 Δ*tcpA* Δ*crp fruB*-FLAG P_{fruB}-*lacZ* Sm^R	This study
JL569	N16961 Δ*tcpA cra*-HA P_{cra}-*lacZ* (-250 → +10) Sm^R	This study
JL576	N16961 Δ*tcpA* Δ*cra fruB*-FLAG P_{fruB}-*lacZ* Sm^R	This study
JL577	N16961 Δ*tcpA* Δ*crp cra*-HA P_{cra}-*lacZ* Sm^R	This study
JL580	N16961 Δ*tcpA* Δ*cra* Δ*crp fruB*-FLAG P_{fruB}-*lacZ* Sm^R	This study
JL581	N16961 Δ*tcpA* Δ*cra* Δ*crp fruB*-FLAG P_{fruB}-*lacZ* pTrc99A::*crp* Sm^RAp^R	This study
JL582	N16961 Δ*tcpA* Δ*cra* Δ*crp fruB*-FLAG P_{fruB}-*lacZ* pTrc99A Sm^RAp^R	This study
JL590	N16961Δ*tcpA fruB*-FLAG P_{fruB_min}-*lacZ* (-50 → +10) Sm^R	This study
JL591	N16961Δ*tcpA* Δ*cra fruB*-FLAG P_{fruB_min}-*lacZ* Sm^R	This study
JL592	N16961Δ*tcpA* Δ*crp fruB*-FLAG P_{fruB_min}-*lacZ* Sm^R	This study
JL597	N16961Δ*tcpA* Δ*cra fruB*-FLAG P_{fruB}-*lacZ* pTrc99A Sm^RAp^R	This study
JL619	N16961Δ*tcpA* Δ*crp fruB*-FLAG P_{fruB}-*lacZ* pJML05 Sm^RAp^R	This study
JL620	N16961Δ*tcpA* Δ*cra* Δ*crp fruB*-FLAG P_{fruB}-*lacZ* pJML05 Sm^RAp^R	This study
JL621	N16961Δ*tcpA* Δ*cra* Δ*crp fruB*-FLAG P_{fruB}-*lacZ* pJML05::*cra* Sm^RAp^R	This study
JL622	N16961Δ*tcpA* Δ*crp fruB*-FLAG P_{fruB_crp}-*lacZ* Sm^R	This study
JL626	N16961Δ*tcpA fruB*-FLAG P_{fruB_crp}-*lacZ* (-93 → +10) Sm^R	This study
JL629	N16961Δ*tcpA fruB*-FLAG P_{fruB_null}-*lacZ* (+11 → +116) Sm^R	This study

Table S1 continued

E. coli		
TOP10	F⁻ *mcrA* Δ(*mrr-hsdRMS-mcrBC*) *Φ*80*lacZ*ΔM15 Δ*lacX74* *recA1 araD139* Δ(*ara leu*)*7697 galU galK rpsL endA1 nupG* Sm^R	Invitrogen
DH5αλpir	F⁻ Δ(*lacZYA-argF*)*U169 recA1 endA1 hsdR17 supE44 thi-1 gyrA96 relA1 λ::pir*	(2)
SM10λpir	*thi recA thr leu tonA lacY supE* RP4-2-Tc::Mu *λ::pir*	(2)
Plasmids		
pCVD442	*oriR6K mobRP4 sacB* Ap^R	(3)
pJL1	pCVD442 derivative with 2.2 kb HpaI-digested VC2338 (*V. cholerae lacZ*) cloned into SmaI site of pCVD442; Ap^R	(4)
pJL1::*lacZ*(*Ec*)	pJL1 derivative with RBS and coding region of *E. coli lacZ* inserted into the VC2338 fragment of pJL1 in an antisense orientation; Ap^R	(5)
pTrc99A	Cloning vector for expression of genes from *trc* promoter; Ap^R	(6)
pTrc99A::*crp*	pTrc99A derivative with coding region from VC2614 (*crp*) inserted between SacI and XbaI sites; Ap^R	This study
pJML05	pTrc99A derivative with the PLlacO-1 promoter in place of the pTrc promoter; Ap^R	This study
pJML05::*cra*	pJML05 derivative with coding region of VCA0519 (*cra*) inserted 50 nt downstream of the start of transcription; Ap^R	This study
pJML05::IGR4	pJML05 derivative in which the IGR4 +1 site directly proceeds the PLlacO-1 promoter	This study

[a]Sm^R, streptomycin resistance; Ap^R, ampicillin resistance. Coordinates of DNA fragments included in *lacZ* fusions are listed in parentheses following the first mention of the fusion. These coordinates are relative to the +1 site of the indicated gene as identified in Papenfort et al., 2015.

Table S2 Primers used in this study

Purpose and primer[a]	Sequence (5' → 3')[b]
Cloning of *V. cholerae fruB*-FLAG	
LIU435 (F1)	GCC AAG CTT GCA TGC CGC GGT TTG TGG TTA GTA GCC
LIU436 (R1)	CCC TTA CTT GTC ATC GTC GTC CTT GTA GTC ACC TTC GCC TAA GCC AGC ATT G
LIU437 (F2)	GAA GGT GAC TAC AAG GAC GAC GAT GAC AAG TAA GGG GCA TCA CAT GAC AAA AAA AGT G
LIU438 (R2)	AGT GAA TTC GAG CTC CGA GTT CGG CGG CGG C
LIU439 (pCVD_R)	TAA CCA CAA ACC GCG GCA TGC AAG CTT GGC GTA ATC ATG
LIU440 (pCVD_F)	CCG CCG CCG AAC TCG GAG CTC GAA TTC ACT GGC CGT
LIU441 (F0)	CAA CTT GAG GTA ATA CTC GCT GG
LIU442 (R0)	CTG CAC CGA CTG TGC TCA C
Cloning of *V. cholerae cra*-HA	
LIU427 (F1)	GCC AAG CTT GCA TGC CAG CGG CTG AAG CTT TAG TCT C
LIU428 (R1)	TGT TTA AGC GTA GTC TGG GAC GTC GTA TGG GTA AGT GCG CAC CTT TAA CTG ACG TG
LIU429 (F2)	ACT TAC CCA TAC GAC GTC CCA GAC TAC GCT TAA ACA AAA TAA AGG TAT GAT ATG CGC CAG
LIU430 (R2)	AGT GAA TTC GAG CTC CGA TGG TCA ACA CGA TCT GAT CC
LIU431 (pCVD_R)	AAG CTT CAG CCG CTG GCA TGC AAG CTT GGC GTA ATC ATG
LIU432 (pCVD_F)	TCG TGT TGA CCA TCG GAG CTC GAA TTC ACT GGC CGT
LIU433 (F0)	CGA AAC GTT ATC AAA CGG GGA TCG
LIU434 (R0)	GCG ACC AAG ATG CCA ATC CG
Cloning of *V. cholerae* Δ*cra*	
LIU446 (F1)	GCC AAG CTT GCA TGC CAG CTG CTT TAG AAT GCC CAA ATG
LIU447 (R1)	CCT TTA TTT TGT TTA CAT AAG GGG GTC TCG TTT TAT GTG
LIU448 (F2)	CGA GAC CCC CTT ATG TAA ACA AAA TAA AGG TAT GAT ATG CGC CAG
LIU449 (R2)	AGT GAA TTC GAG CTC GAT TCA GAC TCC ATC GCG CC
LIU450 (pCVD_F)	GAT GGA GTC TGA ATC GAG CTC GAA TTC ACT GGC CGT

Table S2 continued

LIU451 (pCVD_R)	ATT CTA AAG CAG CTG GCA TGC AAG CTT GGC GTA ATC ATG
LIU452 (F0)	GGA TCA ACG AAG CGT CAA AAT CTG
LIU453 (R0)	GCT GTA TTT CAT CAA TGA GCC AGA G
Cloning of *V. cholerae* P$_{fruB}$-*lacZ*(*Ec*)	
LIU632 (fwd insert)	CAT GGC GTG ATG ATT CGA TGC GGC ATG ATC CGG
LIU633 (rev insert)	GTT TCC TGT GTG AAA AAC CTC GAA TAC TCA CGA TCT TGC
LIU634 (fwd vector)	TGA GTA TTC GAG GTT TTT CAC ACA GGA AAC AGC TAT GAC C
LIU635 (rev vector)	GAT CAT GCC GCA TCG AAT CAT CAC GCC ATG TAT CAG TGG
LIU126 (F0)	GCT GAT CGA CCC GCG CAT AC
LIU127 (R0)	CCA ATG ATC CAC AAT GGG TGA ATG C
Cloning of *V. cholerae* P$_{fruB_\mathrm{min}}$-*lacZ*(*Ec*)	
LIU646 (fwd insert)	CAT GGC GTG ATG ATT ATC CTA GCC TAG TGT TGA ATT ATA CG
LIU647 (rev insert)	GTT TCC TGT GTG AAA GAA TCC TTT CAG CTT TAA TAC TGA ATC G
LIU648 (fwd vector)	AAG CTG AAA GGA TTC TTT CAC ACA GGA AAC AGC TAT GAC C
LIU649 (rev vector)	ACA CTA GGC TAG GAT AAT CAT CAC GCC ATG TAT CAG TGG
LIU126 (F0)	See above
LIU127 (R0)	See above
Cloning of *V. cholerae* P$_{fruB_\mathrm{null}}$-*lacZ*(*Ec*)	
LIU673 (gBlock)	GTT GTC CAC TGA TAC ATG GCG TGA TGA TTA GCA AAA GTA CCG TTG ATT CAC AAT CTC GTC CAC TAC AAA GGT CAG ATT GTG TCG AGT ATC CAG CAG CAA GAT CGT GAG TAT TCG AGG TTT TGC TGA ATT TTT TTG TTT CAC ACA GGA AAC AGC TAT GAC CAT GAT
LIU674 (fwd vector)	TTT CAC ACA GGA AAC AGC TAT GAC C
LIU675 (rev vector)	AAT CAT CAC GCC ATG TAT CAG TGG
LIU126 (F0)	See above
LIU127 (R0)	See above

Table S2 continued

Cloning of *V. cholerae* P$_{fruB_crp}$-*lacZ*(*Ec*)	
LIU679 (gBlock)	GTT TCG TCC ACT GAT ACA TGG CGT GAT GAT TGG GTC TCG TTT TAT GTG CGT CTG ATC ATA GAA CGC GAT TTT TGA TCC TAG CCT AGT GTT GAA TTA TAC GCT GAA TCG ATT CAG TAT TAA AGC TGA AAG GAT TCT TTC ACA CAG GAA ACA GCT ATG ACC ATG AT
LIU674 (fwd vector)	See above
LIU675 (rev vector)	See above
LIU126 (F0)	See above
LIU127 (R0)	See above
Cloning of *V. cholerae* P$_{cra}$-*lacZ*(*Ec*)	
LIU638 (fwd insert)	CAT GGC GTG ATG ATT TGT GAT GTA TCG CTG CAG GTT TTC
LIU639 (rev insert)	GTT TCC TGT GTG AAA ATC CTA GCC TAG TGT TGA ATT ATA CGC
LIU640 (fwd vector)	ACA CTA GGC TAG GAT TTT CAC ACA GGA AAC AGC TAT GAC C
LIU641 (rev vector)	CAG CGA TAC ATC ACA AAT CAT CAC GCC ATG TAT CAG TGG
LIU126 (F0)	See above
LIU127 (R0)	See above
Cloning of pTrc99A::*crp*	
LIU152 (rev vector)	TAT TTT AGC GAA GCC GAG CTC GAA TTC CAT GGT CTG TTT C
LIU153 (fwd vector)	TAC GGC ACT CGC TAA TCT AGA GTC GAC CTG CAG GCA TG
LIU154 (fwd insert)	ATG GAA TTC GAG CTC GGC TTC GCT AAA ATA TGG ATA GCG
LIU155 (rev insert)	CAG GTC GAC TCT AGA TTA GCG AGT GCC GTA AAC CAC G
Cloning of pJML05	
LIU476	TCC GCT CAC ATT TAT CAG CTC ATT TCA GAA TAT TTG CCA GAA C
LIU477	CAA GAT ACT GAC GTC ATG GAA TTC GAG CTC GGT ACC C
LIU480	ATAAATGTGAGCGGATAACATTGACATTGTGAGCGGATAACAAGATACTGACGTC

Table S2 continued

Cloning of pJML05::*cra*	
LIU652 (rev vector)	TGA TCC TAG CCT AGT GAC GTC AGT ATC TTG TTA TCC GCT C
LIU653 (fwd vector)	AAG GTG CGC ACT TAA AAG CTT GGC TGT TTT GGC GGA TG
LIU654 (fwd insert)	CAA GAT ACT GAC GTC ACT AGG CTA GGA TCA AAA ATC GCG
LIU655 (rev insert)	AAA ACA GCC AAG CTT TTA AGT GCG CAC CTT TAA CTG ACG
Cloning of pJML05::IGR4	
LIU494 (fwd vector)	TAT TCG AGG TTT TGC AAG CTT GGC TGT TTT GGC GGA TG
LIU495 (rev vector)	TTG CTG AAT CCT TTC GAC GTC AGT ATC TTG TTA TCC GC
LIU496 (fwd insert)	CAA GAT ACT GAC GTC GAA AGG ATT CAG CAA AAG TAC CGT TG
LIU497 (rev insert)	AAA ACA GCC AAG CTT GCA AAA CCT CGA ATA CTC ACG ATC
cra 5’ RACE	
cra_5’RACE_GSP1	CTA AAG CTT CAG CCG CTG CC
cra_5’RACE_GSP2	GCT TGC CGC GAG TTC TGT TC
M13 Forward	TGT AAA ACG ACG GCC AGT
M13 Reverse	CAG GAA ACA GCT ATG ACC
fruB 5’ RACE	
fruB_5’RACE_GSP1	GGC AGA CAG CAC TCT GGT C
fruB_5’RACE_GSP2	CCG TCT CCC CAA TCC AAA CC
M13 Forward	See above
M13 Reverse	See above
qRT-PCR	
fruB fwd	ATG GGC TTA GCG ACC TTT ATC GC
fruB rev	TCG CGC CAA ATA GCA TAG AGA GTG
fruK fwd	CCT AAC CGA CTG CCA GCA AG
fruK rev	CAG CAT AGA CCA GCA ACC AGC
fruA fwd	ATC ACT GAG GAA ACG ATA GCC GCA
fruA rev	ACT TGA CCA TCG CCA TCC AGG TTA
4.5S fwd	CTG GTC CTC CCG CAA CAC
4.5S rev	GAG ACC CCA GCC ACA TC

[a]fwd, forward; rev, reverse; gBlock, dsDNA fragment; GSP, gene-specific primer.
[b]Underlined regions indicate homology tails for fragment ligation using DNA fragment assembly.

References

1. Papenfort K, Förstner KU, Cong J-P, Sharma CM, Bassler BL. 2015. Differential RNA-seq of *Vibrio cholerae* identifies the VqmR small RNA as a regulator of biofilm formation. Proc Natl Acad Sci 112:E766–E775.

2. Mustachio LML, Aksit SS, Mistry RHR, Scheffler RR, Yamada AA, Liu JM. 2012. The *Vibrio cholerae* mannitol transporter is regulated posttranscriptionally by the MtlS small regulatory RNA. J Bacteriol 194:598–606.

3. Donnenberg MS, Kaper JB. 1991. Construction of an *eae* deletion mutant of enteropathogenic *Escherichia coli* by using a positive-selection suicide vector. Infect Immun 59:4310–4317.

4. Kariisa AT, Grube A, Tamayo R. 2015. Two nucleotide second messengers regulate the production of the *Vibrio cholerae* colonization factor GbpA. BMC Microbiol 15:166.

5. Zhang MG, Liu JM. 2019. Transcription of cis Antisense Small RNA MtlS in *Vibrio cholerae* Is Regulated by Transcription of Its Target Gene, *mtlA*. J Bacteriol 201:e00178-19.

6. Amann E, Brosius J. 1985. "ATG vectors" for regulated high-level expression of cloned genes in *Escherichia coli*. Gene 40:183–190.
